# Supplementary material for: Biologically inspired microlens array camera for high-resolution wide field-of-view imaging
Source: Nat Commun. 2026 Mar 23;17:4343. doi: 10.1038/s41467-026-70967-2 (PMC13172470; doi:10.1038/s41467-026-70967-2)
Supplement: Supplementary file 1 — Supplementary Information [file 41467_2026_70967_MOESM1_ESM.pdf]

Supplementary information for

Biologically inspired microlens array camera for high-resolution wide field-of-view imaging

Jae-Myeong Kwon[1,2], Yejoon Kwon[3], Young-Gil Cha[1,2], Dong Hyun Han[1], Hyun-Kyung Kim[1,2], Je-Kyun Park[1], Min H. Kim[3*], and Ki-Hun Jeong[1,2]*

[1]Department of Bio and Brain engineering, Korea Advanced Institute of Science and Technology (KAIST), 291 Daehak-ro, Yuseong-gu, Daejeon 34141, Republic of Korea.
[2]KAIST Institute for Health Science and Technology (KIHST), Korea Advanced Institute of Science and Technology (KAIST), 291 Daehak-ro, Yuseong-gu, Daejeon, 34141, Republic of Korea.
[3]School of Computing, Korea Advanced Institute of Science and Technology (KAIST), 291 Daehak-ro, Yuseong-gu, Daejeon 34141, Republic of Korea.
Correspondence: Min H. Kim (minhkim@kaist.ac.kr); Ki-Hun Jeong (kjeong@kaist.ac.kr)

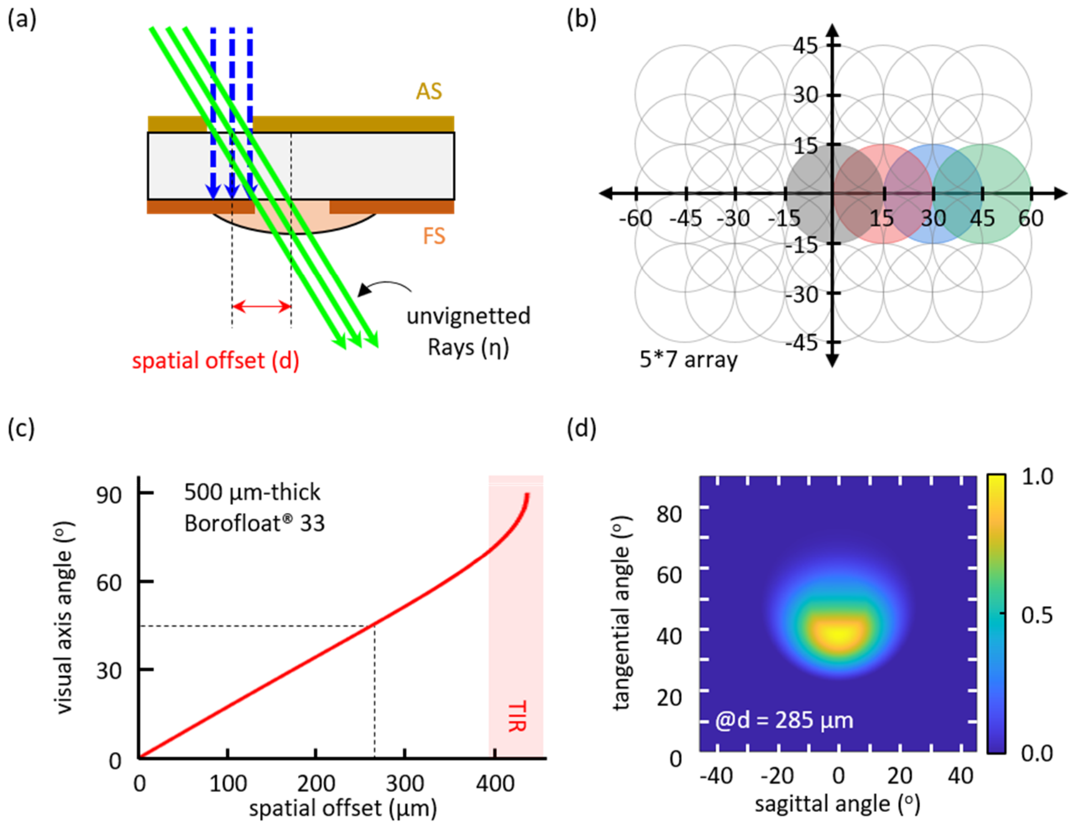

Supplementary Fig. 1: Design of spatially offset-coupled aperture (SOA) for angular sampling. (a) Schematic illustration of a single SOA unit. The upper aperture functions as the aperture stop (AS) that controls the light intensity while the lower aperture acts as the field stop (FS) that limits the acceptance angle. The spatial offset, defined as the lateral distance between the two apertures, determines the visual axis angle of each optical unit. (b) The angular sampling layout of 5 × 7 SOA array. Each unit samples a distinct angular sector to achieve a 140° diagonal FOV through partial overlaps. Adjacent units exhibit approximately 15° overlap, or 40% area for a 30° acceptance angle, which ensures robust multi-view image stitching. (c) Calculated mapping between spatial offset and resulting visual axis angle for a 500 µm-thick Borofloat® 33 substrate. The visual axis angle increases monotonically with offset and can be tuned up to 80° before total internal reflection (TIR) occurs. This monotonic relationship enables precise angular tuning by lithographically adjusting the offset. (d) Angular transmission profile at an offset of 285 µm, indicating peak sensitivity at 45° tangential and 0° sagittal incidence.

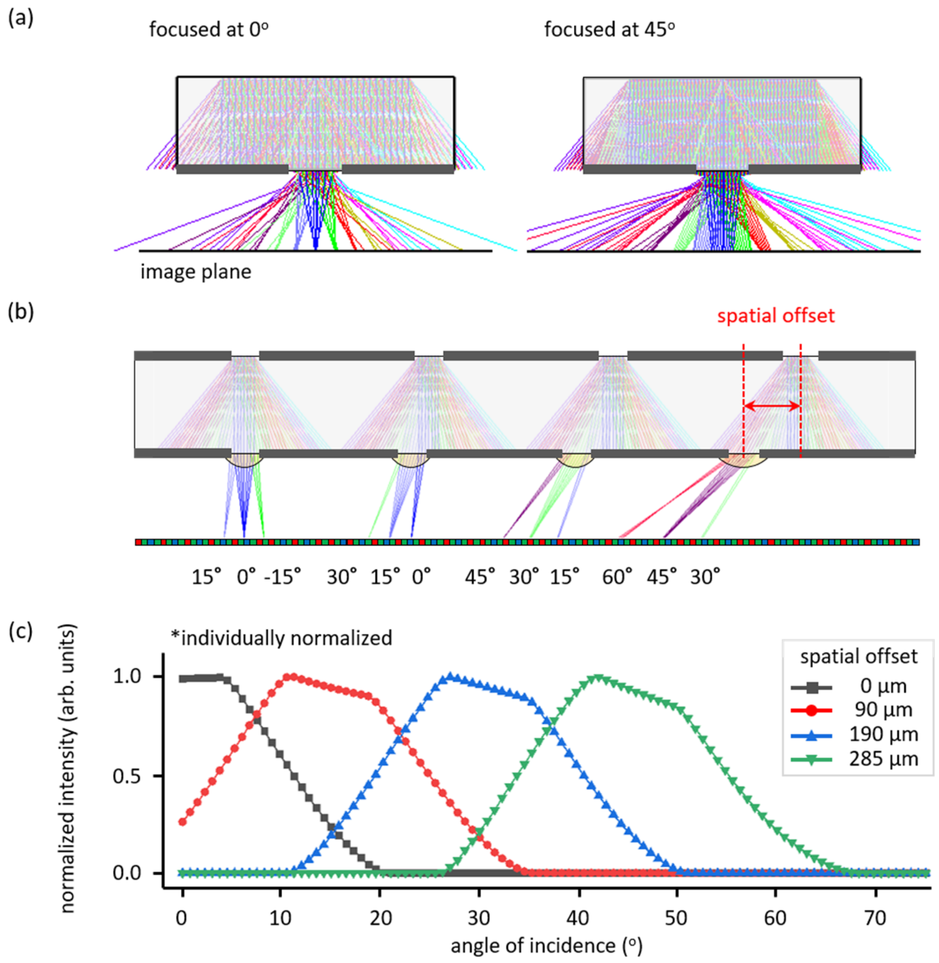

Supplementary Fig. 2: Ray tracing analysis comparing single-layer aperture and SOA configurations. (a) Conventional microlens design with a single-layer aperture. Left: curvature optimized for normal incidence results in defocus and field aberrations at oblique angles. Right: curvature optimized for 45° incidence improves focus for target rays but exhibits blurring at other angles due to insufficient angular selectivity. (b) SOA-based MLA design. Each optical unit transmits light from a designated angular range while rejecting stray rays which provides robust directional filtering. Tailored microlens curvatures correct field-dependent aberrations to support precise focus across a wide FOV. (c) Simulated angular transmission profiles for selected spatial offsets (0, 90, 190, and 285 µm). Each configuration maintains a 30° acceptance angle with peak sensitivity shifted to the desired viewing direction, illustrating tunable and discrete angular sampling.

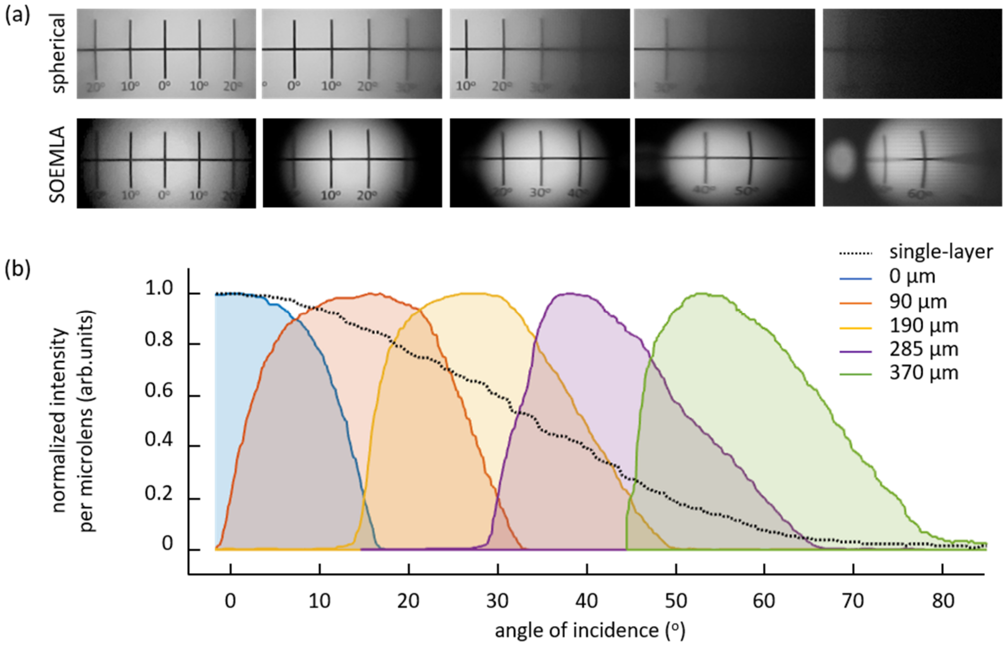

Supplementary Fig. 3: Angular sampling performance of the SOEMLA optical units. (a) Captured images of a FOV test target using a spherical microlens with a single-layer aperture (top row) and the SOEMLAs (bottom row). The conventional setup shows significant brightness and contrast loss beyond 30°, caused by off-axis aberrations and vignetting. In contrast, the SOEMLAs capture discrete partial images at specific viewing angles with high brightness and contrast through angular filtering by SOAs. (b) Measured angular sensitivity profiles of individual SOEMLA optical units with different spatial offsets. Each unit exhibits a sharply peaked transmission curve centered at its designated incidence angle, demonstrating effective angular compartmentalization. In comparison, spherical microlenses without angular filtering (dotted line) show broad and non-selective responses.

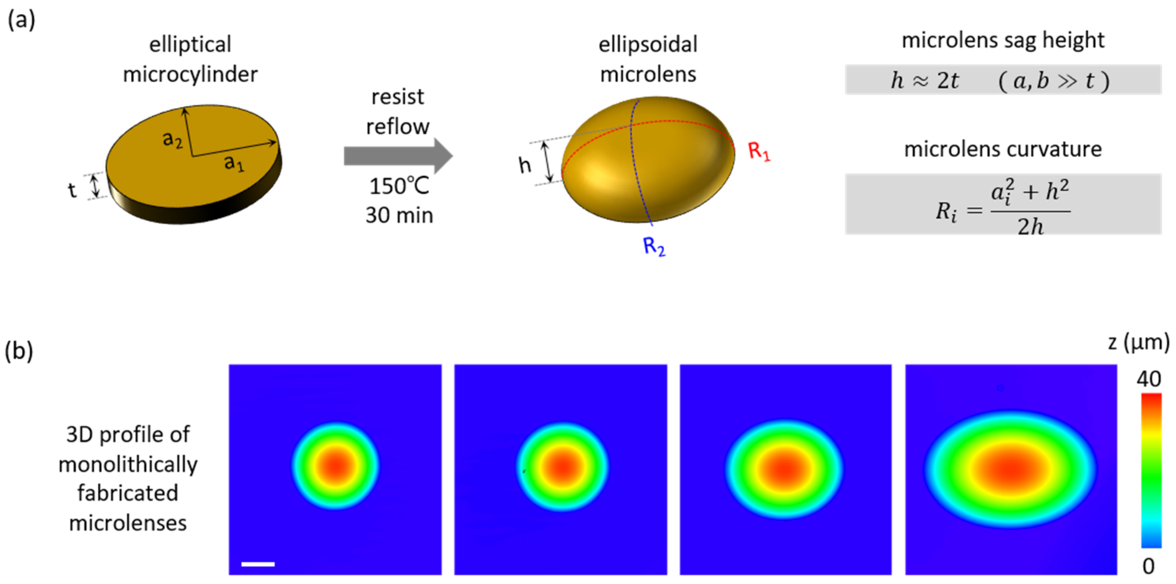

sag height	38.4	38.4	38.8	39.3
major axis length	280	295	375	540
minor axis length	280	285	315	370
major axis ROC	292.9	324.3	505.5	1041
minor axis ROC	292.6	302.9	364.2	493.5

unit: μm

Supplementary Fig. 4: Monolithic fabrication of ellipsoidal microlenses via resist reflow. (a) Schematic of thermal reflow process. Elliptical microcylinders with axis dimensions (a_1, a_2) and thickness t reflow into ellipsoidal microlenses at 150**°C**. The sag height remains constant ($h \approx 2t$) while ROC along each axis is determined by the corresponding axis length by a_i. (b) Confocal 3D profiles of monolithically fabricated microlenses on a single 4-inch wafer. Lithographic variation of axis dimensions results in a wide range of asymmetric curvatures and demonstrates precise geometric control. The fabricated microlenses maintain exceptional uniformity across the wafer. The average sag height is 39.04 μm with a standard deviation of 0.59 μm, which reflects highly reproducible ellipsoidal curvature formation through the resist reflow process. Scale bar: 100 μm.

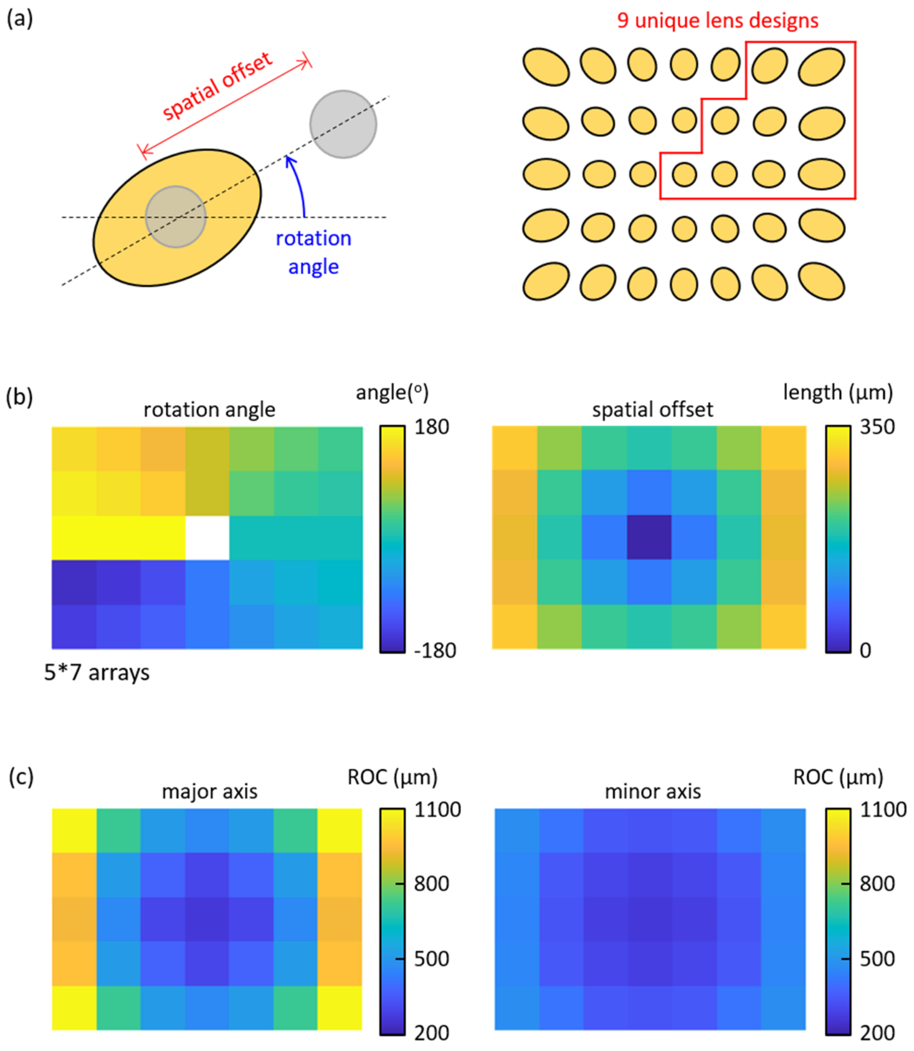

Supplementary Fig. 5: Final design parameters of the 5 × 7 SOEMLA optical units. (a) Schematic illustrating the spatial offset and microlens rotation angle used to define the angular acceptance of each optical unit. Nine unique optical units are used with varying rotations. (b) Heatmaps showing the rotation angle (left) and spatial offset (right) assigned to each optical unit. The offset increases radially to achieve wider angular coverage, while the rotation ensures correct directional alignment. (c) Design parameters of the ellipsoidal microlens curvature. The ROC is separately defined for the major (left) and minor (right) axes. Units with larger incidence angles have higher curvature and greater anisotropy, compensating for field curvature and astigmatism. Note that units responsible for wider viewing angles exhibit increasing total curvature and anisotropy.

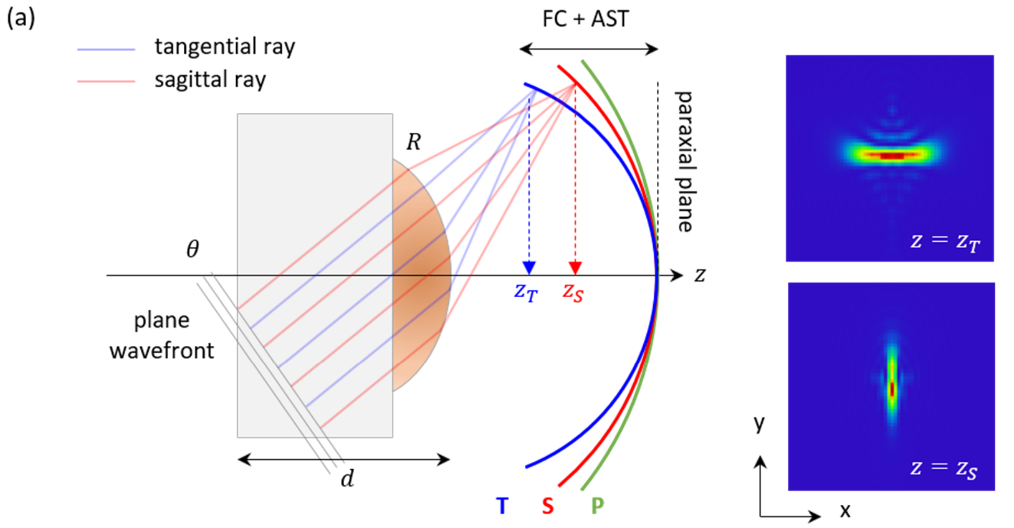

$$z_S = EFL - \frac{EFL\,\theta^2}{2}\left[\frac{1}{n_l} + 1 - \frac{(d+2R)d}{R^2}\theta^2\right], \quad z_T = EFL - \frac{EFL\,\theta^2}{2}\left[\frac{1}{n_l} + 3\left\{1 - \frac{(d+2R)d}{R^2}\theta^2\right\}\right]$$

FC S. AST FC T. AST

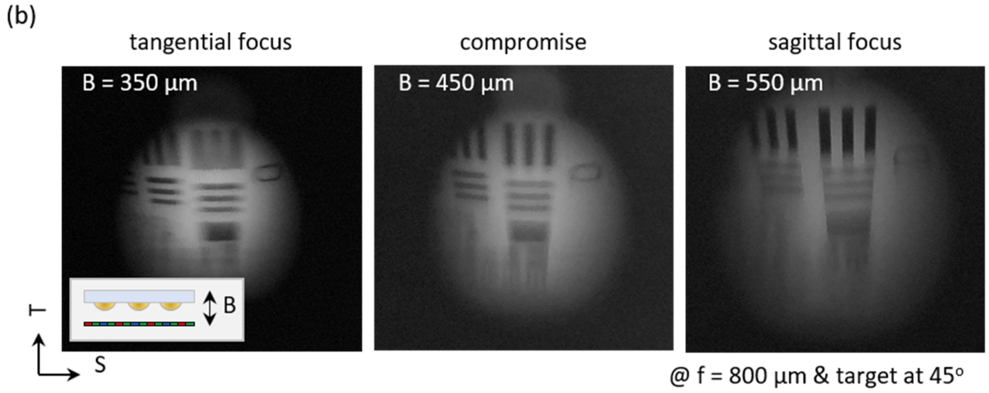

Supplementary Fig. 6: Field curvature and astigmatism at wide incidence angles. (a) Geometric origin of field curvature (FC) and astigmatism (AST) in a single plano-convex microlens. Asymmetric convergence of sagittal (red) and tangential (blue) rays creates two distinct focal planes, z_S and z_T, leading to astigmatic blur. Calculated point spread functions (PSFs) at each focal plane (right) exhibit characteristic elongation patterns. Other aberrations such as coma and spherical aberration are excluded from this analysis, as their contributions are negligible compared to dominant FC and AST at large incidence angles with sufficiently small aperture sizes. (b) Experimental validation using spherical MLA cameras with different image distances (B) for a target located at a 45° field angle. A sensor position at the tangential focus (B = 350 μm) results in dominant sagittal blur, while placement at the sagittal focus (B = 550 μm) causes tangential blur. An intermediate distance (B = 450 μm) produces a compromise image with reduced overall contrast.

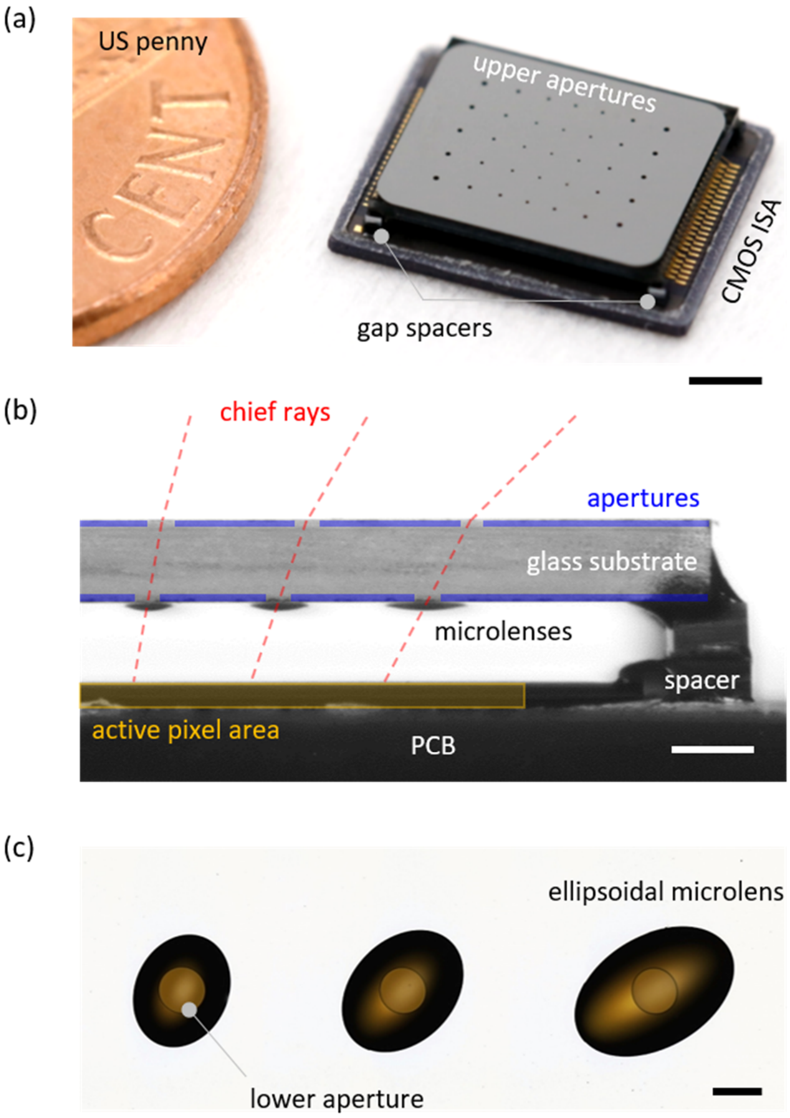

Supplementary Fig. 7: Optical images of the fully packaged SOEMLA camera. (a) Top view of the fully assembled SOEMLA camera showing the upper apertures, gap spacers, and CMOS ISA. Scale bar: 2 mm. (b) Cross-sectional optical image of the microfabricated SOEMLAs integrated with the CMOS ISA. The upper and lower apertures (120 nm thick) are not visible in the optical image and are indicated by color overlays to denote the positions. As the incidence angle increases, the spatial offset and microlens size increase correspondingly to maintain directional imaging. Scale bar: 500 µm. (c) Close-up images of individual microlenses verify high optical quality and precise alignment, achieved through the resist reflow fabrication process. Scale bar: 100 µm. The complete SOEMLA camera delivers high-resolution, wide FOV imaging within a total track length of less than 1 mm.

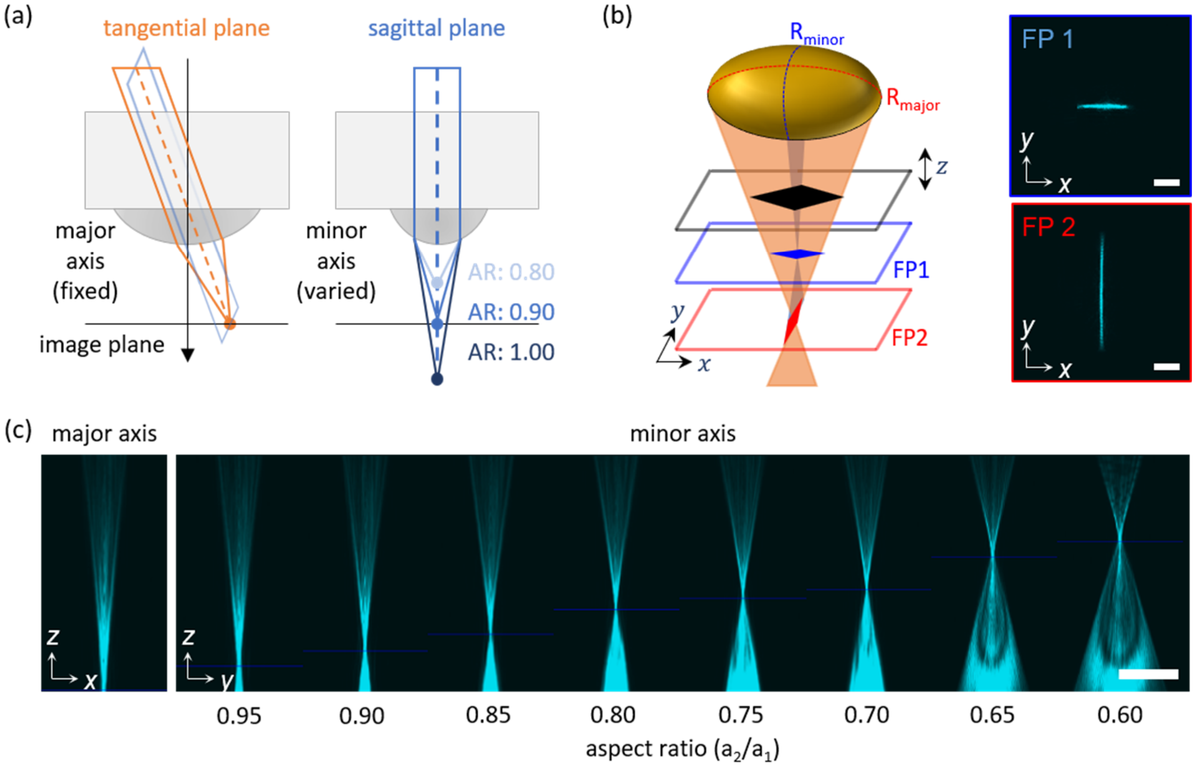

Supplementary Fig. 8: Asymmetric focusing behavior of ellipsoidal microlenses. (a) Schematic illustration of ray propagation in the tangential (left) and sagittal (right) planes for an ellipsoidal microlens. The curvature of the major axis is fixed to maintain focus in the tangential plane, while the minor axis curvature is varied to tune sagittal focus. Depending on the aspect ratio (AR), sagittal rays converge at different depths relative to the image plane, influencing astigmatic performance. (b) Confocal laser scanning setup (left) used to measure focal lengths along orthogonal axes by capturing axial spot profiles. Acquired focal plane images (right) show the sagittal focus (FP1) and tangential focus (FP2), confirming distinct focal positions and validating anisotropic optical behavior of the ellipsoidal design. Scale bars: 10 µm. (c) Confocal laser scanning microscopy results showing axial focal sectioning for ellipsoidal microlenses with different aspect ratios. The major-axis focal length was fixed at 800 µm, while the minor-axis curvature was varied. Scale bar: 200 µm.

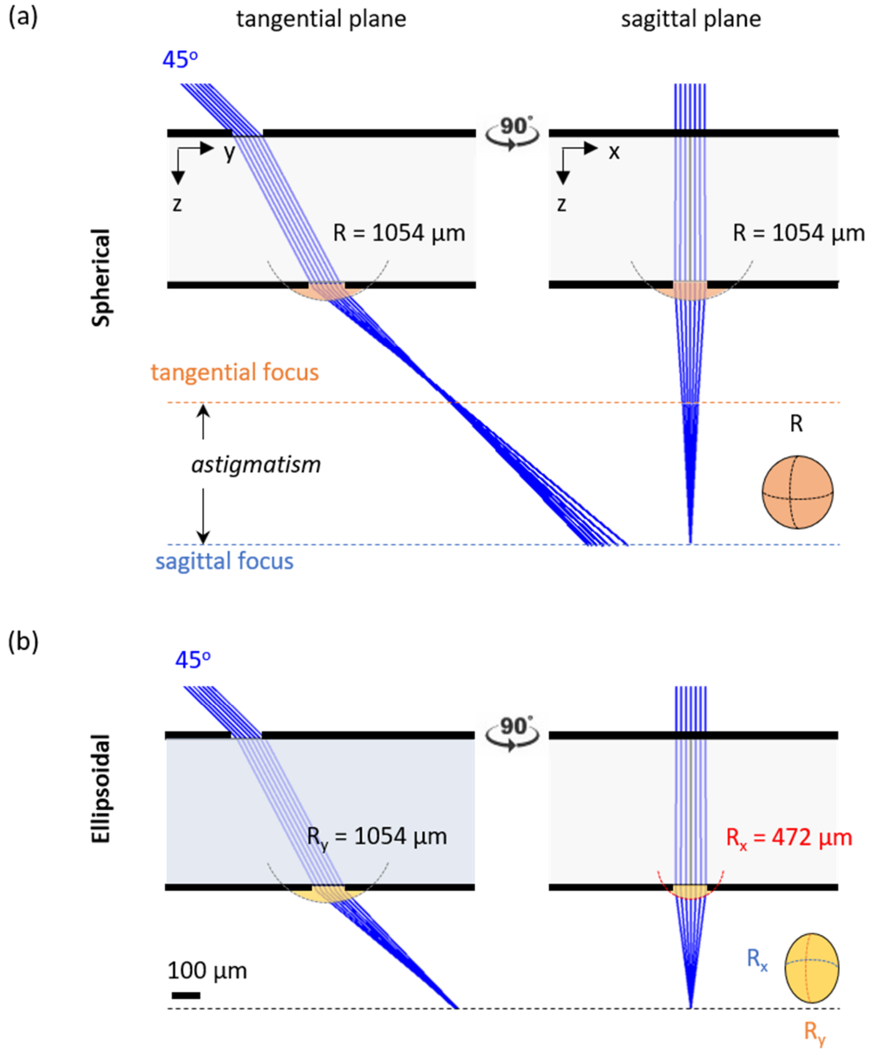

Supplementary Fig. 9: Ray tracing analysis of microlens curvature optimization. (a) Spherical microlens with a fixed curvature (R = 1054 µm) focusing light incident at 45°. The sagittal and tangential planes suffer from distinct focal planes due to astigmatism. (b) Ellipsoidal microlens with independently optimized sagittal (R_x = 472 µm) and tangential (R_y = 1054 µm) curvatures. The asymmetric design compensates for off-axis aberrations, aligning sagittal and tangential focal points to minimize spot size. The curvature values were derived using Zemax simulations and individually applied based on each optical unit's designated viewing angle. The integration of angle-specific ellipsoidal microlenses with the angular filtering provided by SOAs facilitates high-resolution imaging across a wide angular range.

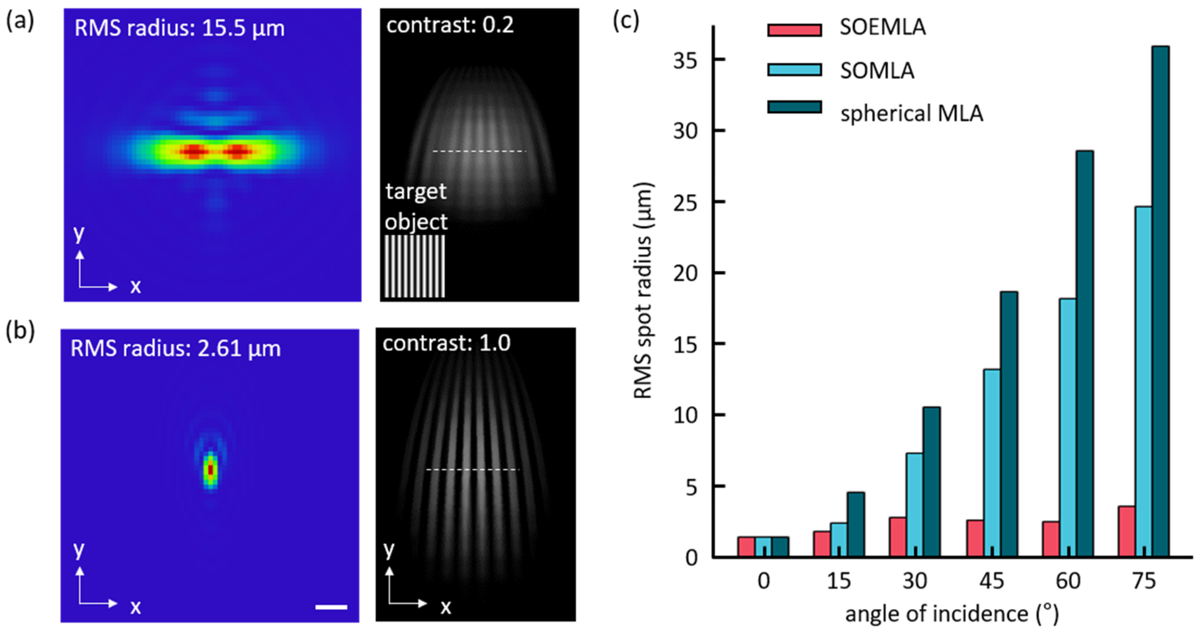

Supplementary Fig. 10: Comparison of PSFs and RMS spot sizes for different microlens configurations. Simulated PSFs and images of (a) a spherical microlens and (b) an ellipsoidal microlens under 45° incidence. The spherical microlens shows sagittal elongation of the PSF (RMS radius: 15.5 μm) due to astigmatism, resulting in blurring and low image contrast (0.2). In contrast, the ellipsoidal microlens tailored for 45° incidence produces a symmetric PSF (RMS radius: 2.61 μm) and restores image contrast to 1.0. Scale bar: 10 μm. (c) RMS spot radius plotted against angle of incidence for three configurations: spherical microlenses, spherical microlenses with SOA filtering (SOMLA), and ellipsoidal microlenses with SOA (SOEMLA). While spherical designs degrade rapidly at high angles, the SOEMLA configuration maintains consistently small spot sizes across the full angular range, confirming its effectiveness in correcting wide-angle aberrations.

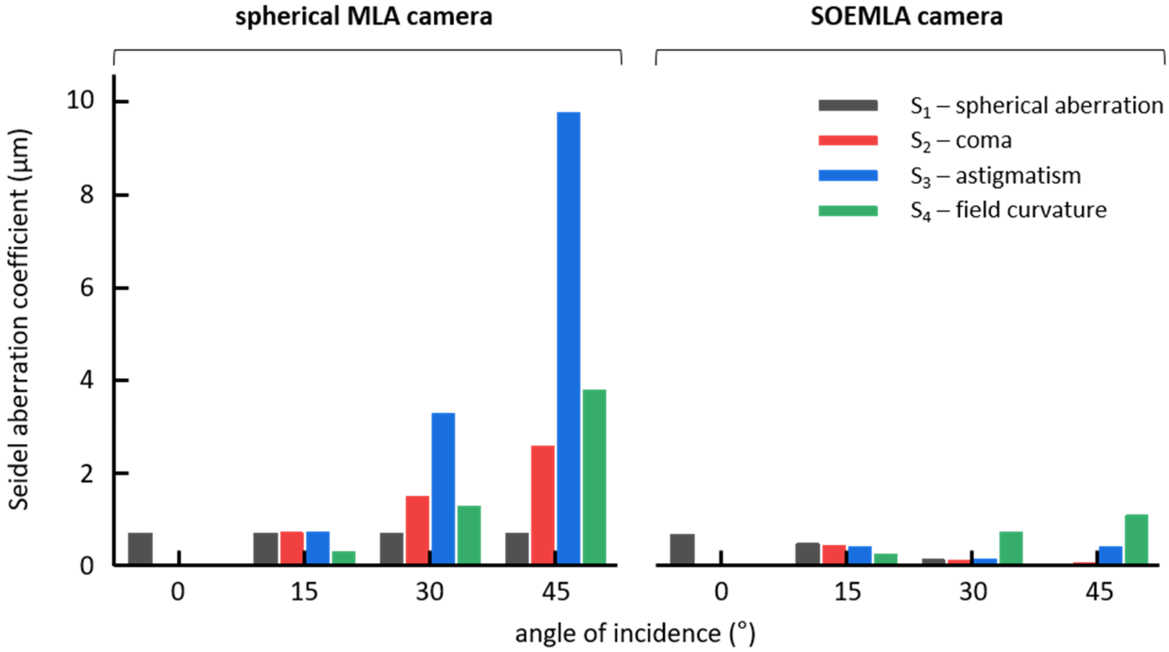

Supplementary Fig. 11: Calculated Seidel aberration coefficients (S_1–S_4) as a function of incidence angle for the spherical MLA and SOEMLA cameras. The coefficients correspond to S_1 spherical aberration, S_2 coma, S_3 astigmatism, and S_4 field curvature. While the spherical MLA exhibits rapidly increasing astigmatism and field curvature at large angles (30°–45°), the SOEMLA maintains uniformly low aberrations across all angles, confirming effective suppression of angular-dependent aberrations in simulation.

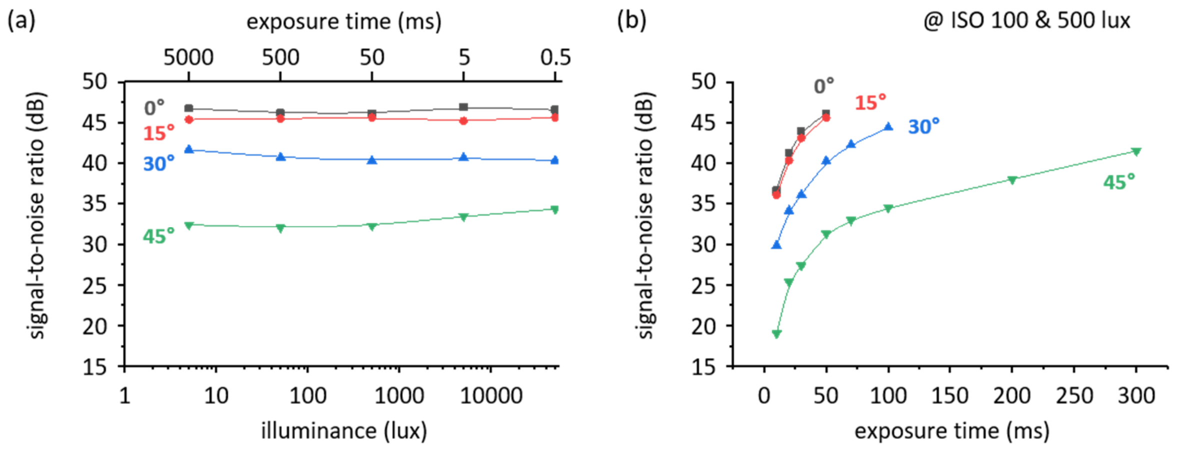

Supplementary Fig. 12: Measured signal-to-noise ratio (SNR) of the SOEMLA camera. (a) SNR across illuminance levels (5 – 50,000 lux) for visual-axis angles of 0°, 15°, 30°, and 45°. All measurements were performed at ISO 100 with the ISP disabled using linear RAW data. Exposure time was inversely scaled with illuminance to maintain a constant photon budget. The results show that single-shot imaging maintains practical SNR levels from central to peripheral views, despite the expected reduction at oblique visual-axis angles. (b) SNR versus exposure time at a fixed illuminance of 500 lux, measured under the ISO 100, ISP-off, linear RAW conditions. Longer exposure compensates for the angular throughput loss at oblique views, which leads to consistent SNR performance across the FOV in the multi-exposure measurements.

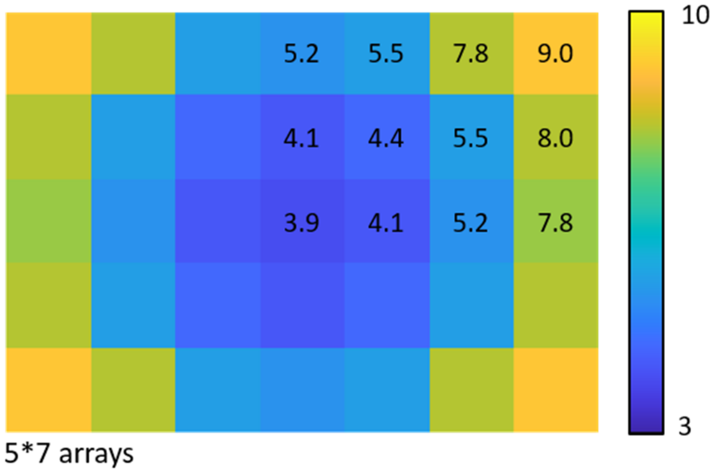

Supplementary Fig. 13: Effective f-number distribution across the optical units of the SOEMLA camera. The effective f-number for each unit accounting for oblique incidence was computed using ZEMAX ray tracing. The measured variation in photon throughput across units shows good agreement with the optical model. For example, the ~12 dB SNR reduction at 45° observed in Fig. S12 is consistent with the combined loss from the increased effective f-number ($20\log_{10}[3.9/7.8] \approx -6$ dB) and the cosine-law irradiance falloff ($20\log_{10}[\cos^2 45°] \approx -6$ dB).

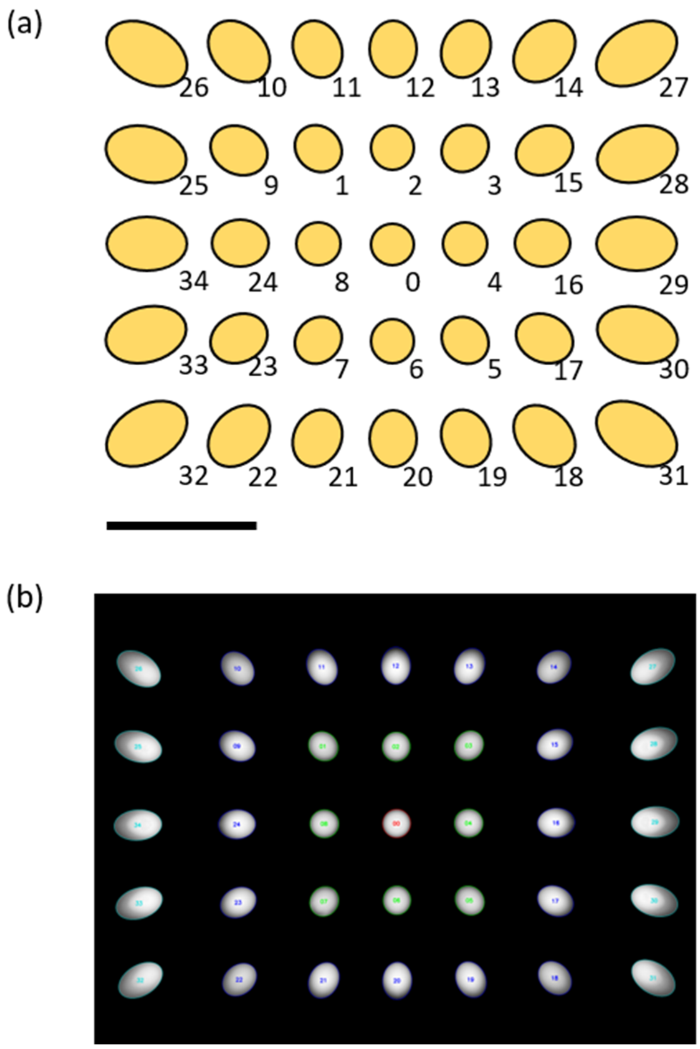

Supplementary Fig. 14: Optical unit labeling and partial FOV masking of the SOEMLA camera. (a) Layout and relative dimensions of the 35 ellipsoidal microlenses arranged in a 5 × 7 array. Each optical unit is spirally labeled for image stitching and calibration. Scale bar: 1 mm. (b) Partial images extracted from a captured white reference image. The distinct shapes and positions of the masked regions reflect direction-specific angular sampling by each optical unit.

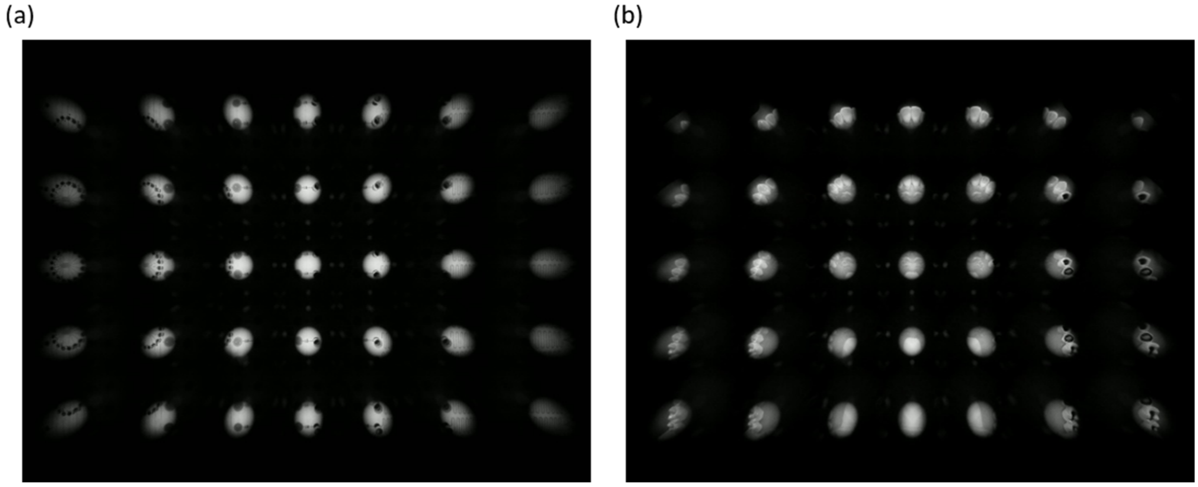

Supplementary Fig. 15: Raw array images captured by the SOEMLA camera. Captured images of (a) the large-area microfluidic chip and (b) the full-scale dental phantom corresponding to the wide FOV reconstructed images shown in Fig. 5. The Cr aperture layer absorbs approximately 40% of incident light upon each reflection, and sufficient inter-unit pitch ensures optical isolation that minimizes stray light and ghosting artifacts in the raw images. The optical noise between partial images is approximately 20 dB lower than the signal level.

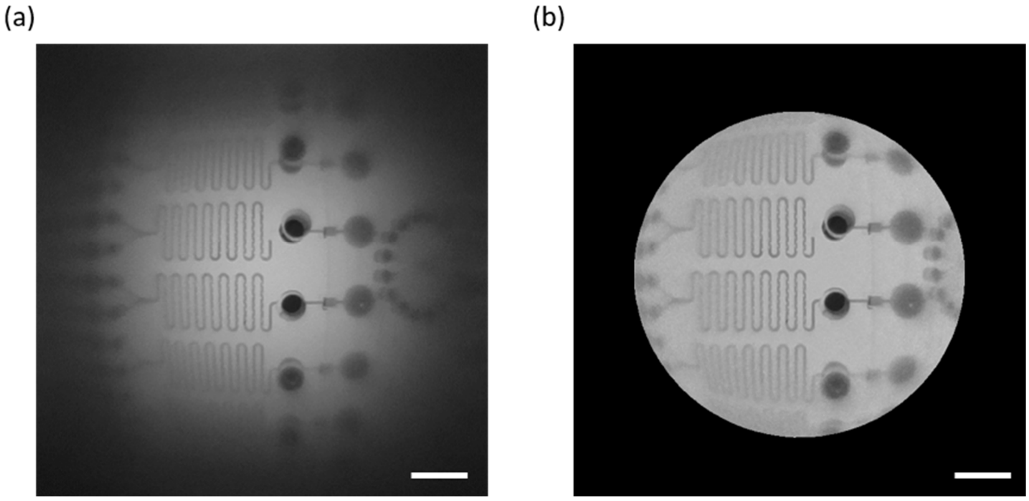

Supplementary Fig. 16: Image correction result for the spherical MLA camera. (a) Raw large-area microfluidic chip image. (b) Reconstructed image after applying the lens-shading and distortion corrections. Spherical MLA cameras are limited to a much narrower effective image processing region compared to SOEMLA cameras, owing to significant aberrations and vignetting. Scale bars: 5 mm.

Supplementary Table 1: Detailed microfabrication steps of SOEMLAs.

Fig. #	Process	Material	Condition
I	bottom-side lift-off coating	DNR-L300-D1	2 μm thickness
	bottom-side e-beam evaporation	Cr	120 nm thickness
	lift-off	DPS-7300	gentle ultrasonication
	top-side lift-off coating	DNR-L300-D1	2 μm thickness
	top-side e-beam evaporation	Cr	120 nm thickness
	lift-off	DPS-7300	gentle ultrasonication
II	top-side PR deposition	DNR-L4615D	24 μm thickness
III	thermal reflow	-	convection oven<br>150°C, 30 min
IV	spacer bonding	alumina spacer,<br>UV-curable epoxy	440 μm image distance

Supplementary Table 2: Processing time for wide FOV stitching.

Stage	Process	Avg. time per unit (sec)	Avg. time per image (sec)
camera calibration	ROI detection	0.03435	0.8938
	lens shading correction	1.494	3.913
	distortion correction	25.15	40.85
	homography matching	5.603	12.43
image reconstruction	warping (homography)	0.3759	1.585
	simple blending	-	8.568
	Poisson blending	-	46.48

Supplementary Table 3: Comparison of recent ultrathin wide FOV camera

representative works	platform	wide FOV strategy	system height	FOV	image quality
Y. M. Song et al. [1]	curved MLA	hemispheric detector	~ 6 mm	160.4°	180 ommatidia ($\Delta\Phi$ = 11.0°)
D. Floreano et al. [2]	curved MLA	hemispheric detector	~ 12.8 mm	180°	630 ommatidia ($\Delta\Phi$ = 4.2°)
Y. Zhou et al. [3]	curved pinhole array	hemispheric detector	~ 20 mm	143°	121 ommatidia ($\Delta\Phi$ = 10°)
B. Dai et al. [4]	curved MLA + planar sensor	optical waveguides	~ 2.5 mm (+ 50 mm optional lens)	170°	522 ommatidia ($\Delta\Phi$ ~ 8.0°)
Z.-Y. Hu et al. [5]	curved MLA + planar sensor	logarithmic microlenses	TTL < 1mm	90°	19 – 160 ommatidia (R ~ 0.08 MP)
Y. Liu et al. [6]	Metalens	AI correction	1.57 mm	140°	PSNR: 15.296 dB SSIM: 0.65916
J. Chen et al. [7]	Metalens	angle-specific nanostructure	~ 3 mm	120°	up to 191 lp/mm at sensor
A. Martins et al. [8]	Metalens	quadratic phase profile	f ~ 750 µm	170°	resolution ~ 2 λ_0 (focusing efficiency: 3.5%)
D. Keum et al. [9]	planar MLA	microprism	TTL: 1.4 mm	68°	up to 181 lp/mm at sensor
J.-M. Kwon et al. [this work]	planar MLA	offset aperture ellipsoidal lens	TTL: 0.94 mm	140°	PSNR: 20.09 dB SSIM: 0.793 (R ~ 1 MP)

*interommatidial angle: $\Delta\Phi$
**image resolution after reconstruction

Supplementary Note 1: Hyperfocal distances of the SOEMLA camera

The hyperfocal distance (H) defines the closest distance at which a lens can be focused while keeping objects at infinity acceptably sharp. When the image sensor is focused at H, the depth-of-field (DOF) extends from $H/2$ to infinity, and when H becomes shorter than the typical object distance, the system effectively exhibits an infinite DOF.

The hyperfocal distance is given by

$$H = \frac{f^2}{Nc} + f,$$

where f is the focal length of the microlens, N is the f-number, and c is the permissible circle of confusion (CoC).

Adopting a diffraction-limited criterion for visible light, we set the CoC to the Airy-disk diameter $c = 2.44\,\lambda N$ (with $\lambda = 550$ nm), which yields $H \leq 9.93$ mm based on the SOEMLAs' focal length and f-numbers.

This result indicates that the SOEMLA camera maintains focus from approximately 5 mm to infinity, thereby achieving an effectively infinite DOF across the entire FOV.

Supplementary Note 2: Pixel economy for wide FOV imaging using the SOEMLA camera

The pixel economy (E) is defined as the ratio between the total number of pixels in the fully stitched image ($N_{stitched}$) and that of the image sensor (N_{sensor}):

$$E = \frac{N_{stitched}}{N_{sensor}} = \frac{F}{R}$$

where F and R denote the sensor fill factor and partial image overlap redundancy, respectively. R is calculated from the number of microlenses (M_x, M_y) and the overlap ratios between adjacent partial images (k_x, k_y) as follows:

$$R = \frac{1}{1 - k_x(1 - 1/M_x)} \cdot \frac{1}{1 - k_y(1 - 1/M_y)}$$

Note that a larger F improves E but increases the likelihood of optical crosstalk between optical units, while a smaller R (mainly lower k) enhances E but reduces the robustness of image stitching.

In the SOEMLA camera design, the theoretical pixel economy

$$E\big(F = 0.2, M_x = 7, M_y = 5, k_x = k_y = 0.4\big) \approx 0.1$$

corresponds to a stitched resolution of 1 MP.

The fill factor F cannot be arbitrarily increased, as a larger aperture or a shorter lens pitch induces optical crosstalk and stray-light leakage between adjacent microlenses. The fill factor was set to $F = 0.2$ to suppress noise in the double-layer structure. Further improvement of F is possible through multi-layer aperture designs or the use of light-absorbing layers that mitigate stray light.

Similarly, the overlap ratio k cannot be arbitrarily reduced since too small overlap leads to unstable image registration and mismatch. A minimum $k \geq 0.10$ (corresponding to $R \geq 1.2$) is generally required for robust stitching. In the fabricated SOEMLA camera, geometric calibration and lens-level correction were incorporated; therefore, the overlap ratio was set to $k = 0.4$ to ensure robust stitching performance under various imaging conditions.

Supplementary References

1. Song, Y. M. et al. Digital cameras with designs inspired by the arthropod eye. *Nature* **497**, 95-99 (2013).
2. Floreano. D. et al. Miniature curved artificial compound eyes. *Proceedings of the National Academy of Sciences* **110**, 9267-9272 (2013).
3. Zhou, Y. et al. An ultrawide field-of-view pinhole compound eye using hemispherical nanowire array for robot vision. *Science Robotics* **9**, eadi8666 (2024).
4. Dai, B. et al. Biomimetic apposition compound eye fabricated using microfluidic-assisted 3D printing. *Nature communications* **12**, 6458 (2021).
5. Hu, Z.-Y. et al. Miniature optoelectronic compound eye camera. *Nature Communications* **13,** 5634 (2022).
6. Liu, Y. et al. Ultra-wide FOV meta-camera with transformer-neural-network color imaging methodology. *Advanced Photonics* **6**, 056001-056001 (2024).
7. Chen, J. et al. Planar wide-angle-imaging camera enabled by metalens array. *Optica* **9**, 431-437 (2022).
8. Martins, A. et al. On metalenses with arbitrarily wide field of view. *ACS Photonics* **7**, 2073-2079 (2020).
9. Keum, D. et al. Xenos peckii vision inspires an ultrathin digital camera. *Light: Science & Applications* **7**, 80 (2018).
