# Supplementary material for: Biologically inspired microlens array camera for high-resolution wide field-of-view imaging
Source: Nat Commun. 2026 Mar 23;17:4343. doi: 10.1038/s41467-026-70967-2 (PMC13172470; doi:10.1038/s41467-026-70967-2)
Supplement: Supplementary file 2 — Description Of Additional Supplementary File [file 41467_2026_70967_MOESM2_ESM.pdf]

Description of Additional supplementary files

Supplementary Data 1:

Representative raw images for camera calibration and wide field-of-view imaging. This dataset includes representative raw images used in the processing pipeline, covering (1) lens shading correction, (2) distortion correction, (3) homography estimation and geometric alignment, and (4) wide field-of-view image capture.

Supplementary Movie 1:

Wide FOV video capture using the SOEMLA camera. The SOEMLA camera successfully captures the surrounding environment with a 280 mm × 160 mm FOV on the 50 mm targets from an 80 mm working distance.
