# Supplementary figures and images for: Biologically inspired microlens array camera for high-resolution wide field-of-view imaging
Source: Nat Commun. 2026 Mar 23;17:4343. doi: 10.1038/s41467-026-70967-2 (PMC13172470; doi:10.1038/s41467-026-70967-2)

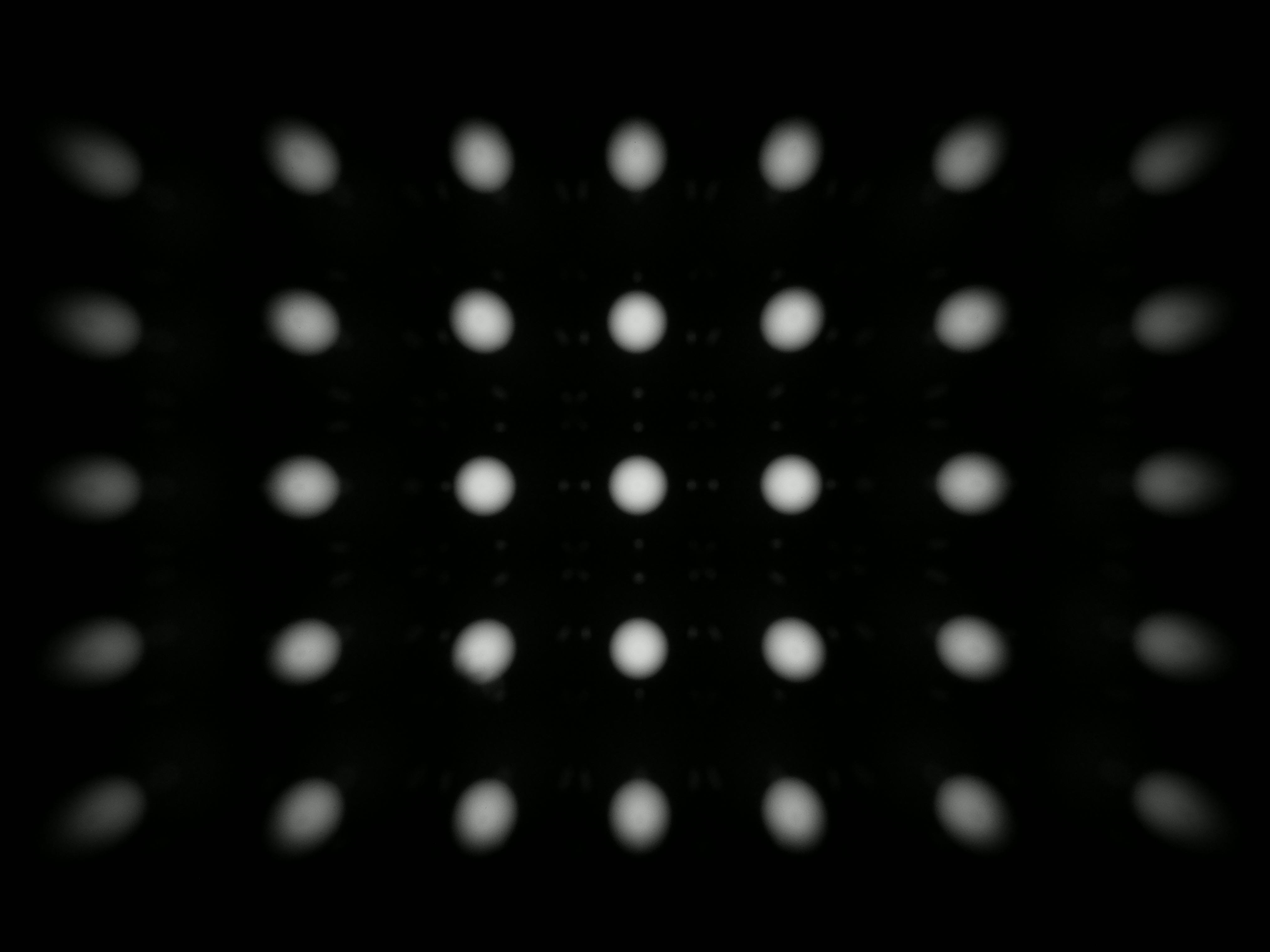

Supplement: Supplementary file 3 — Supplementary Data 1 [file 41467_2026_70967_MOESM3_ESM.zip › 1. lens shading correction/white_01.tiff]

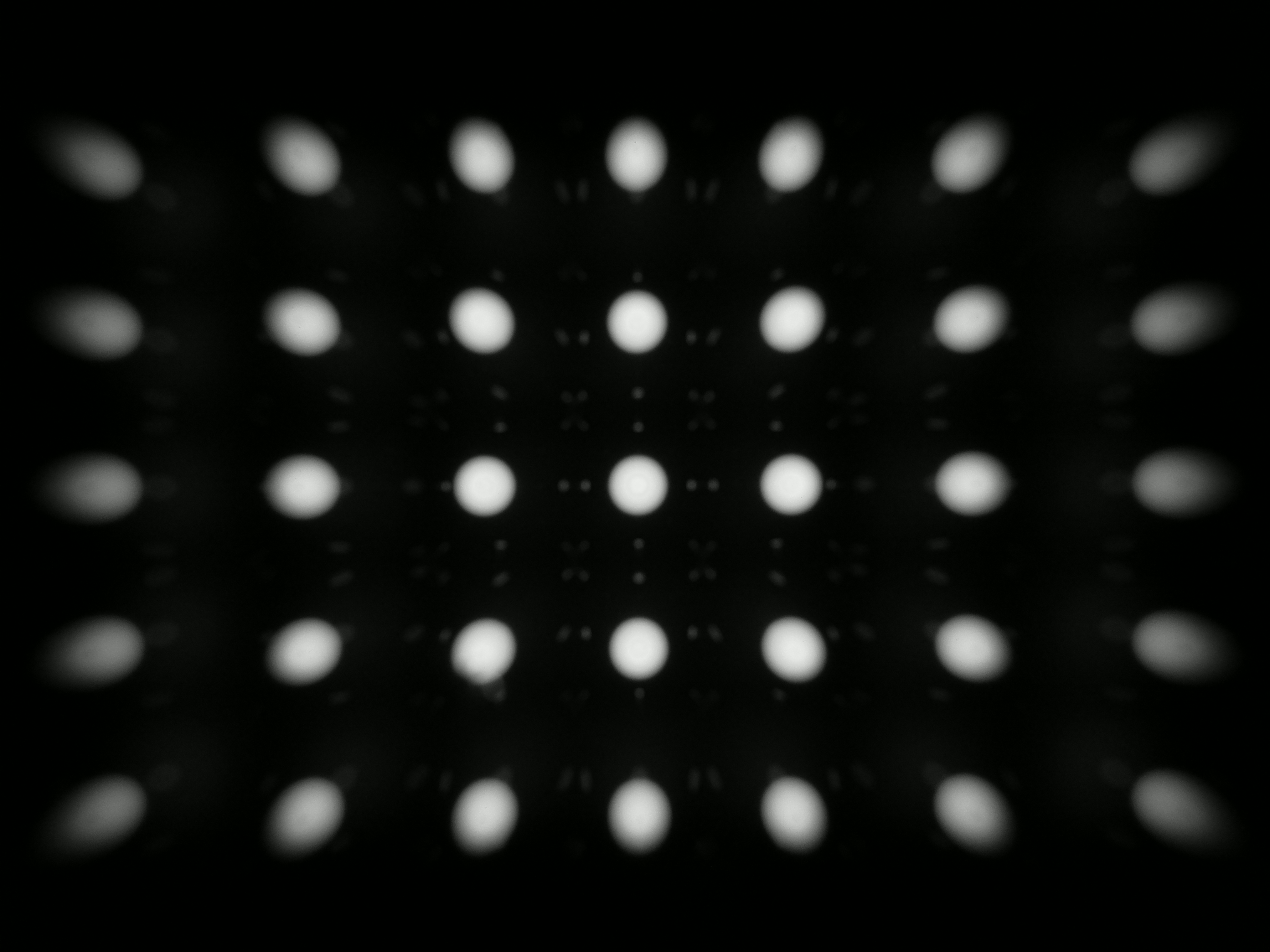

Supplement: Supplementary file 3 — Supplementary Data 1 [file 41467_2026_70967_MOESM3_ESM.zip › 1. lens shading correction/white_02.tiff]

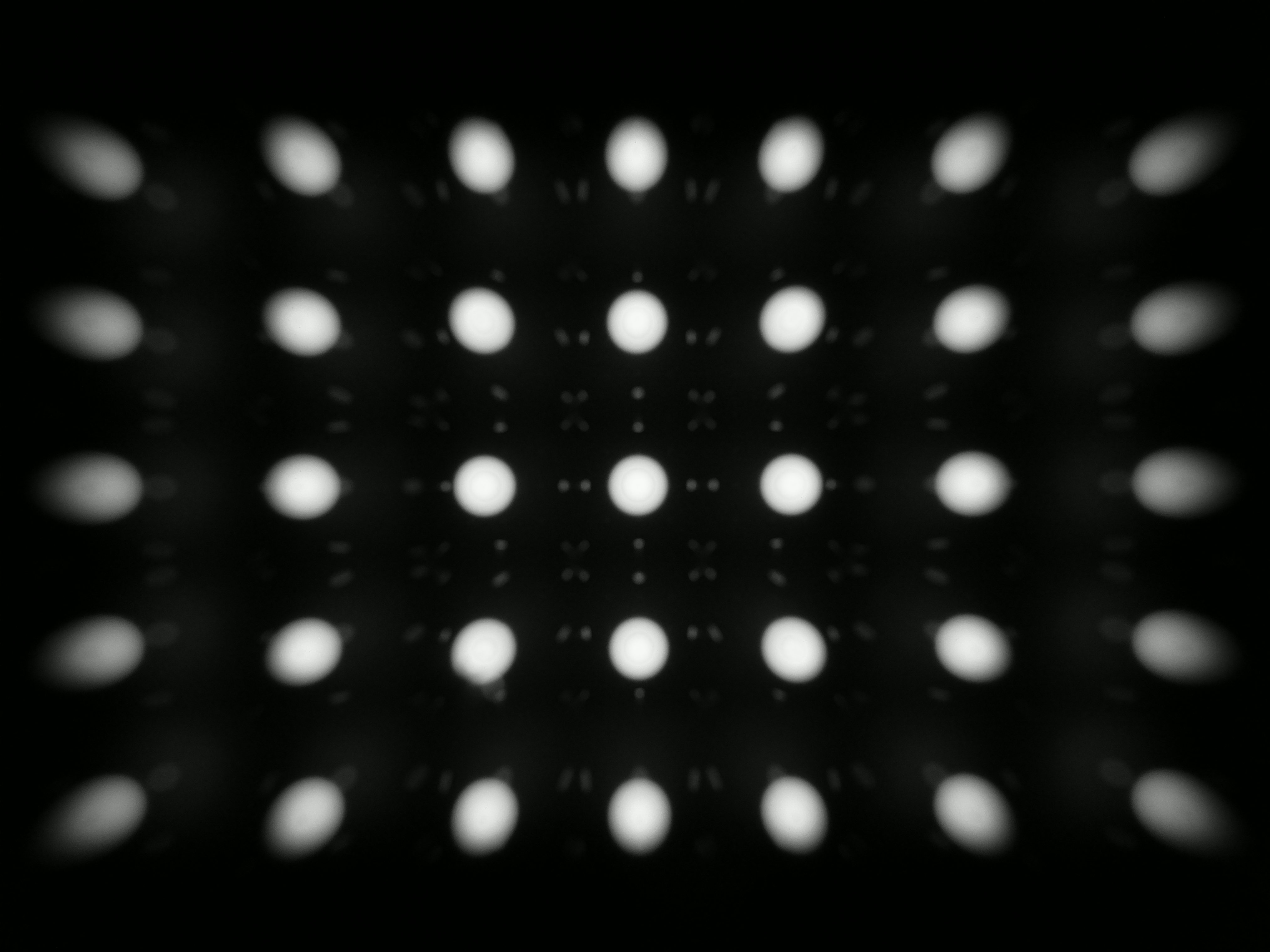

Supplement: Supplementary file 3 — Supplementary Data 1 [file 41467_2026_70967_MOESM3_ESM.zip › 1. lens shading correction/white_03.tiff]

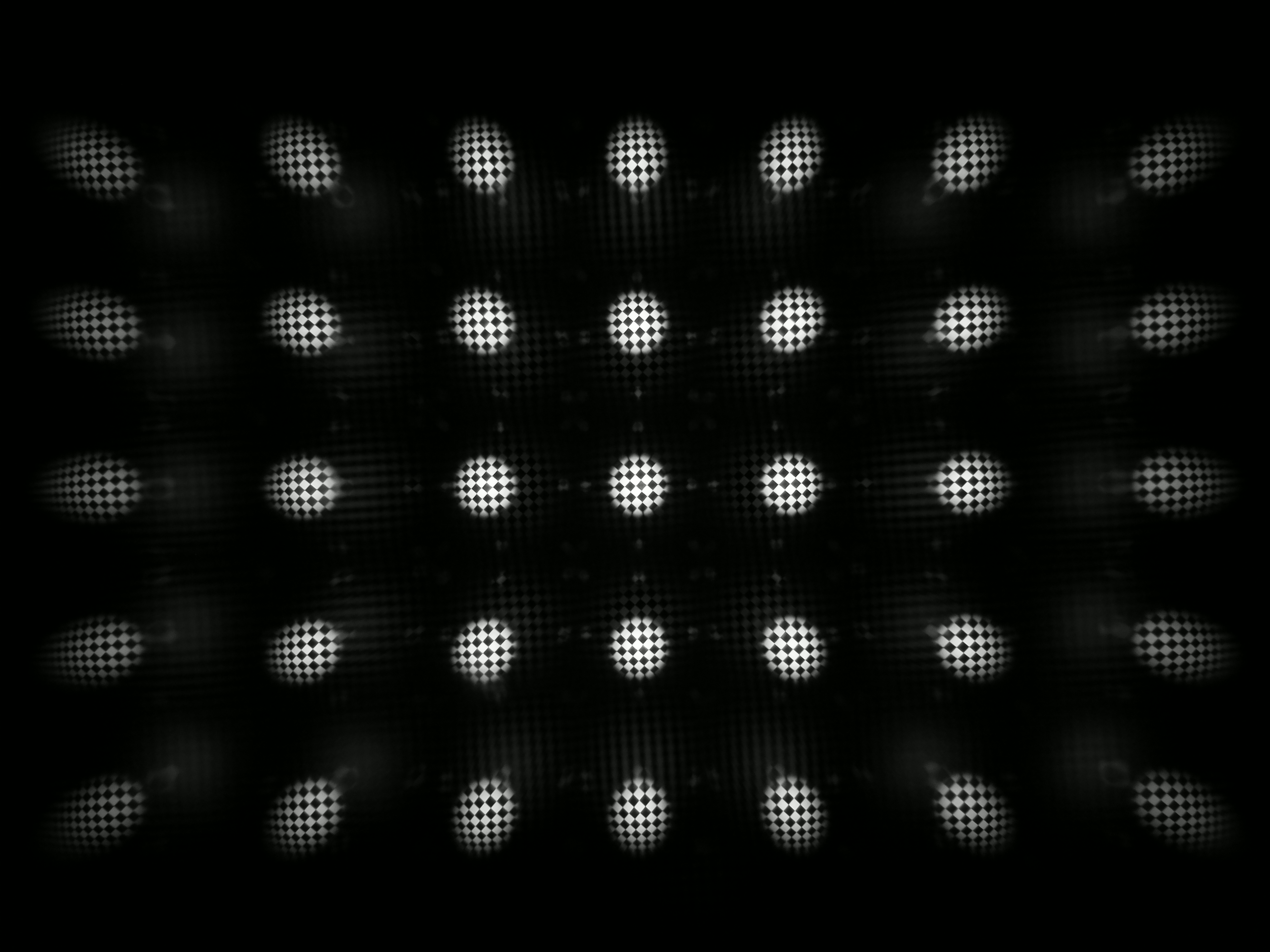

Supplement: Supplementary file 3 — Supplementary Data 1 [file 41467_2026_70967_MOESM3_ESM.zip › 2. distortion correction/chess_000.tiff]

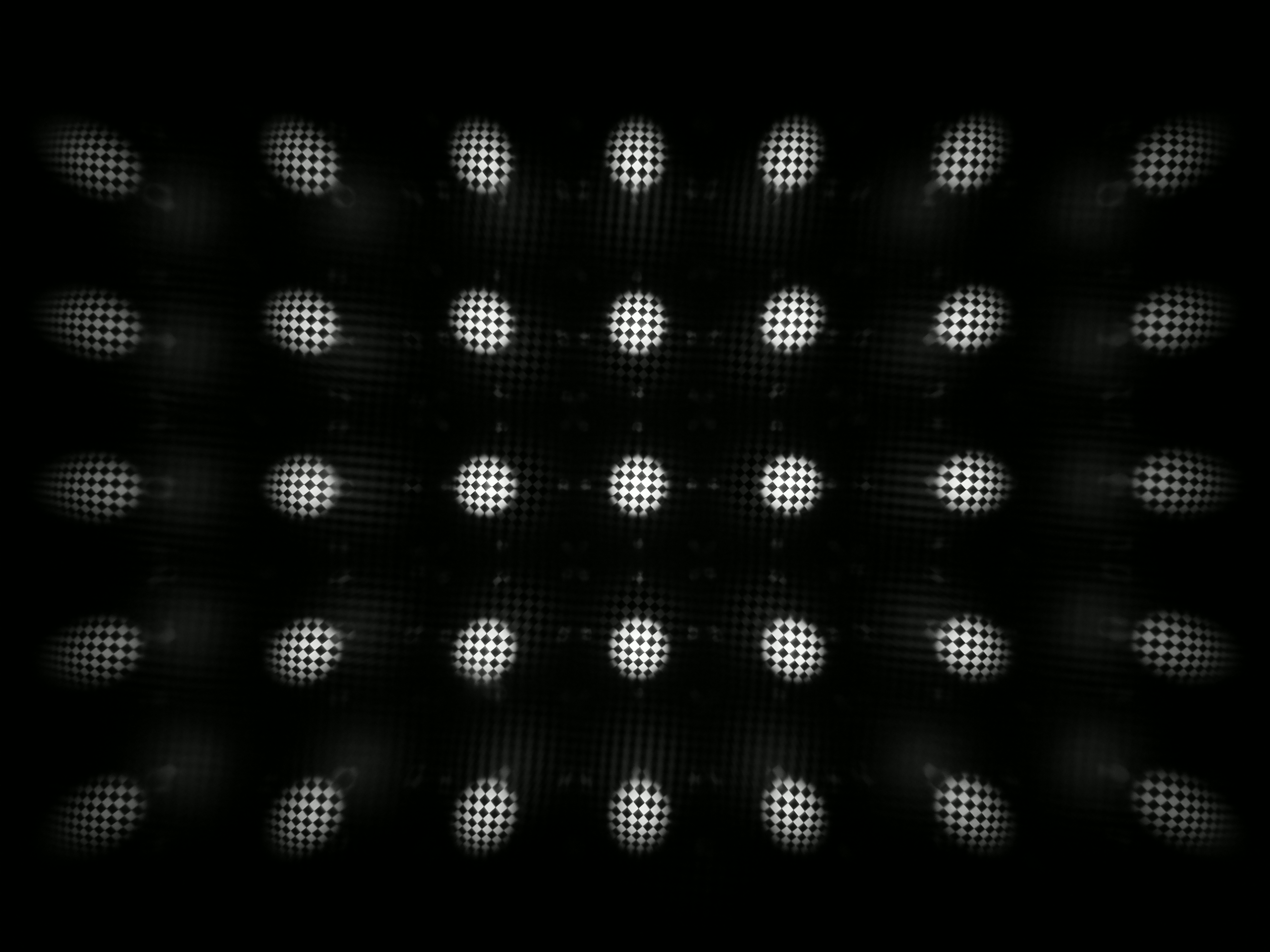

Supplement: Supplementary file 3 — Supplementary Data 1 [file 41467_2026_70967_MOESM3_ESM.zip › 2. distortion correction/chess_001.tiff]

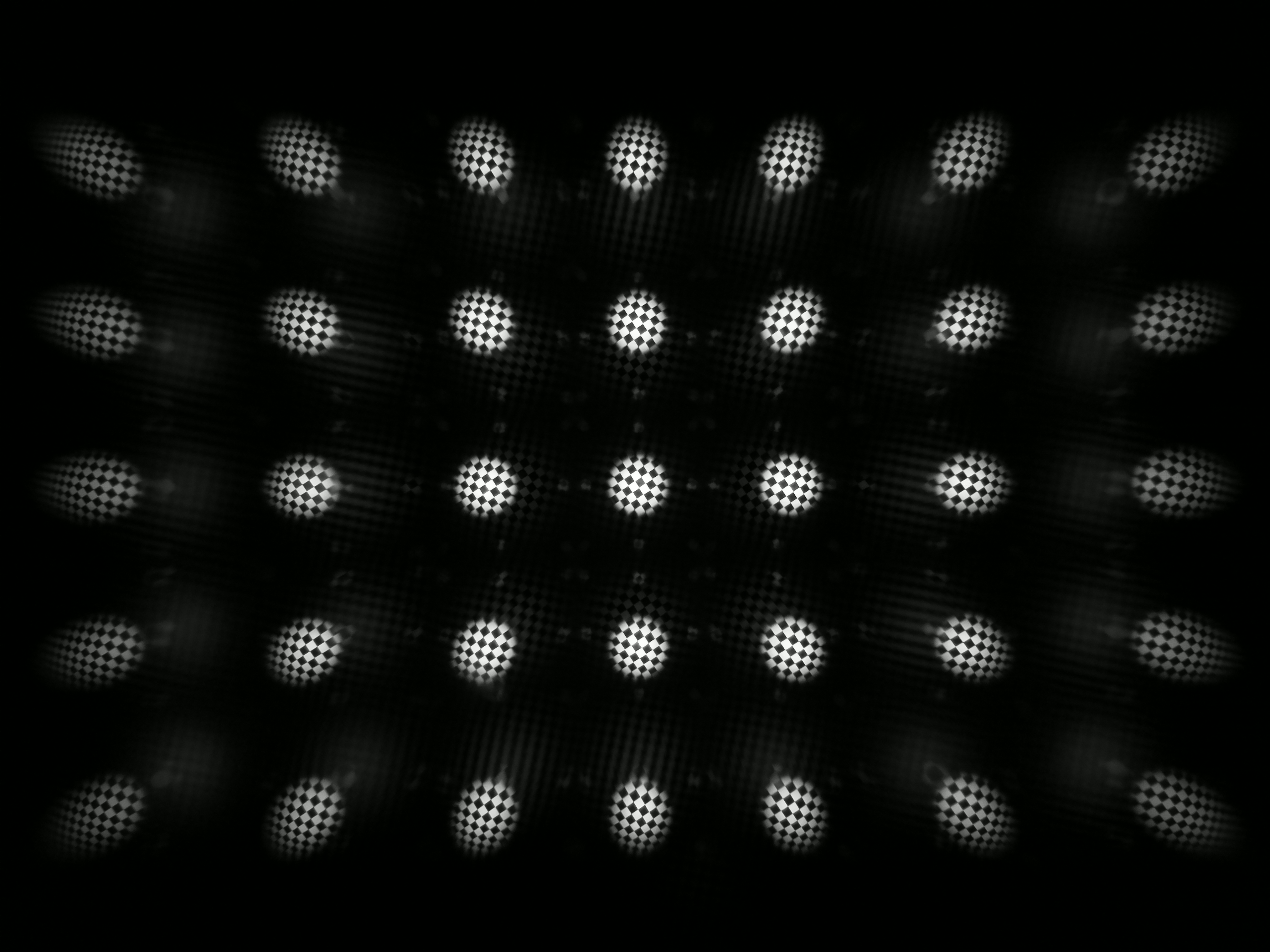

Supplement: Supplementary file 3 — Supplementary Data 1 [file 41467_2026_70967_MOESM3_ESM.zip › 2. distortion correction/chess_002.tiff]

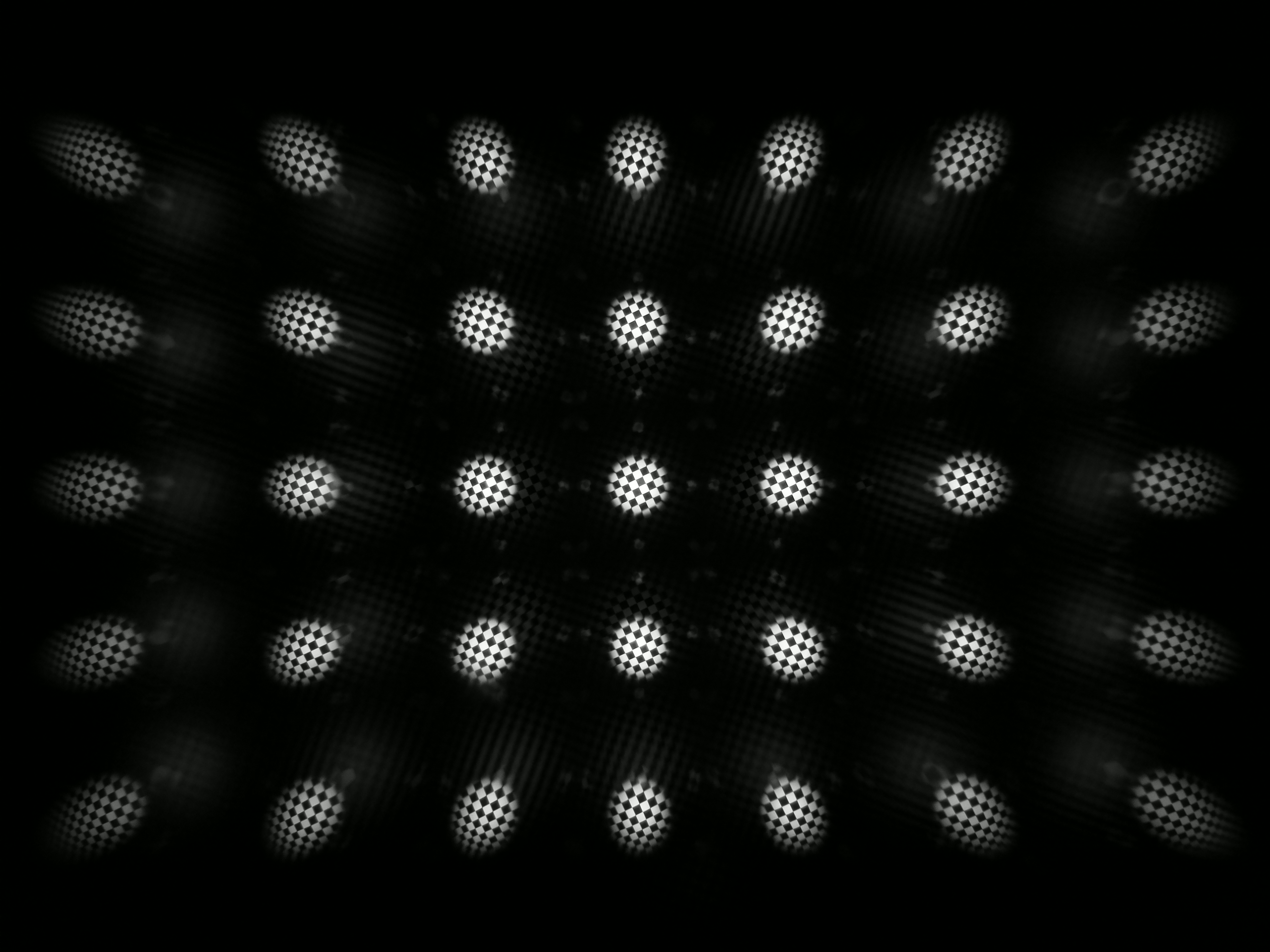

Supplement: Supplementary file 3 — Supplementary Data 1 [file 41467_2026_70967_MOESM3_ESM.zip › 2. distortion correction/chess_003.tiff]

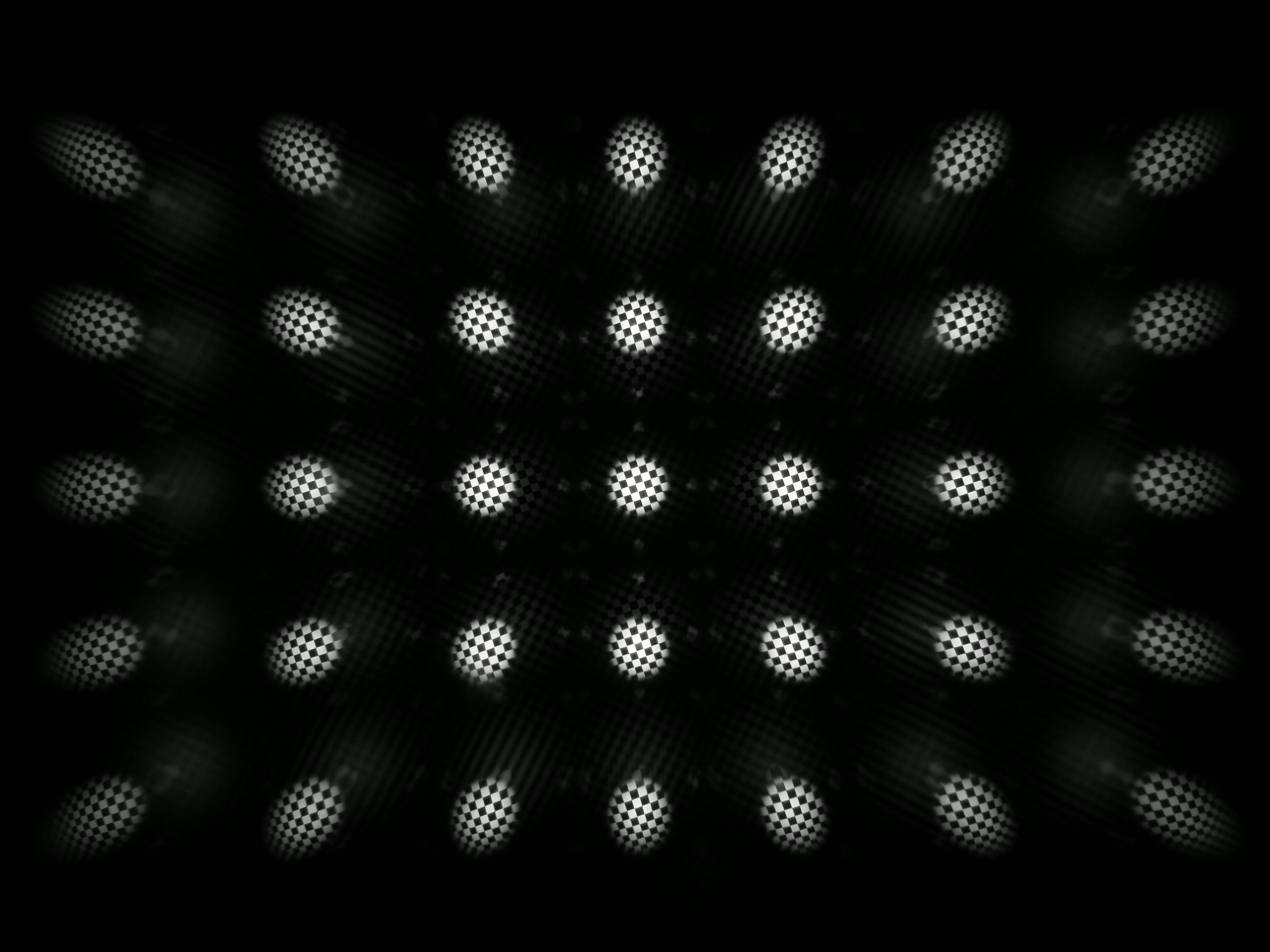

Supplement: Supplementary file 3 — Supplementary Data 1 [file 41467_2026_70967_MOESM3_ESM.zip › 2. distortion correction/chess_004.tiff]

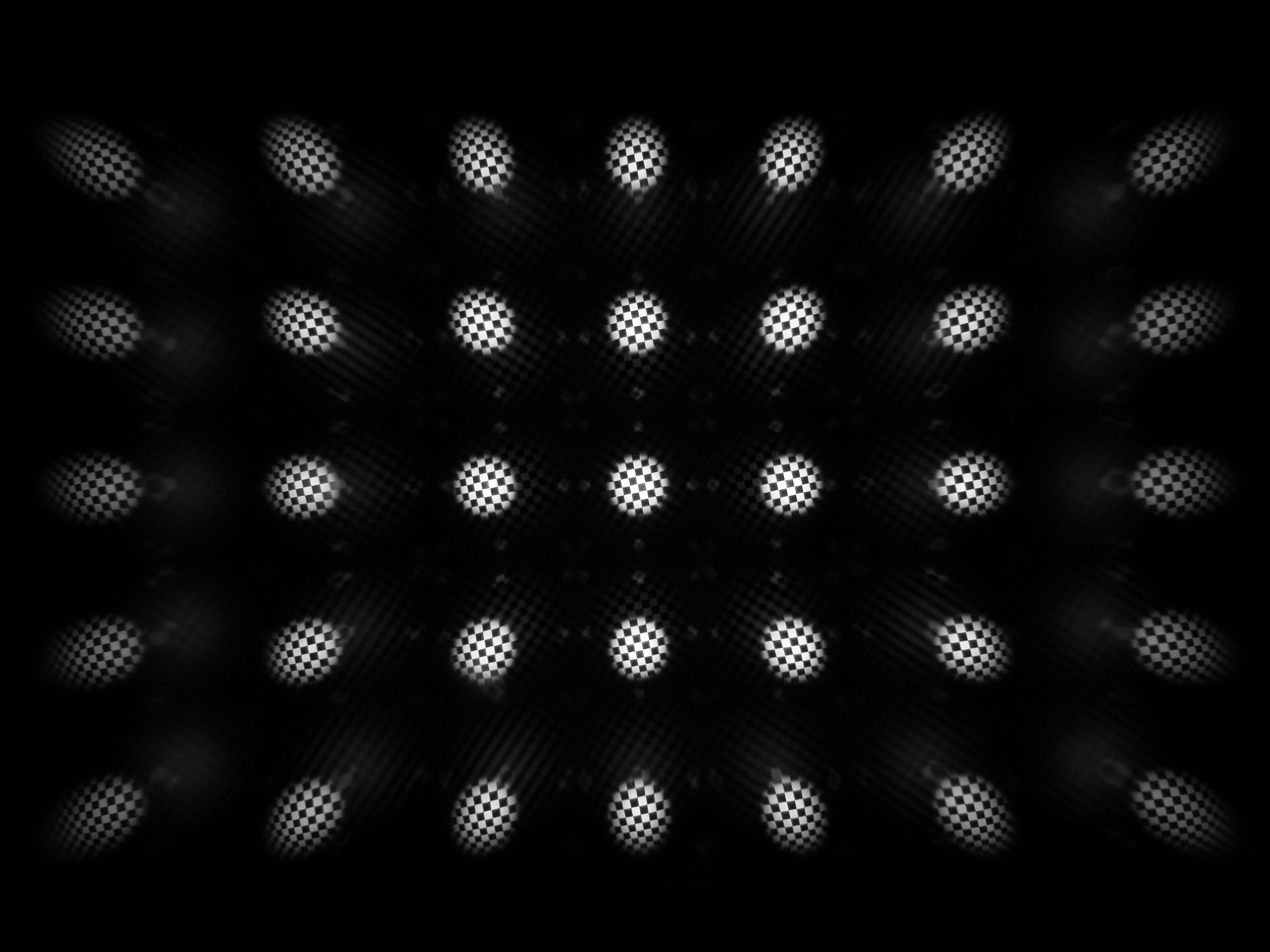

Supplement: Supplementary file 3 — Supplementary Data 1 [file 41467_2026_70967_MOESM3_ESM.zip › 2. distortion correction/chess_005.tiff]

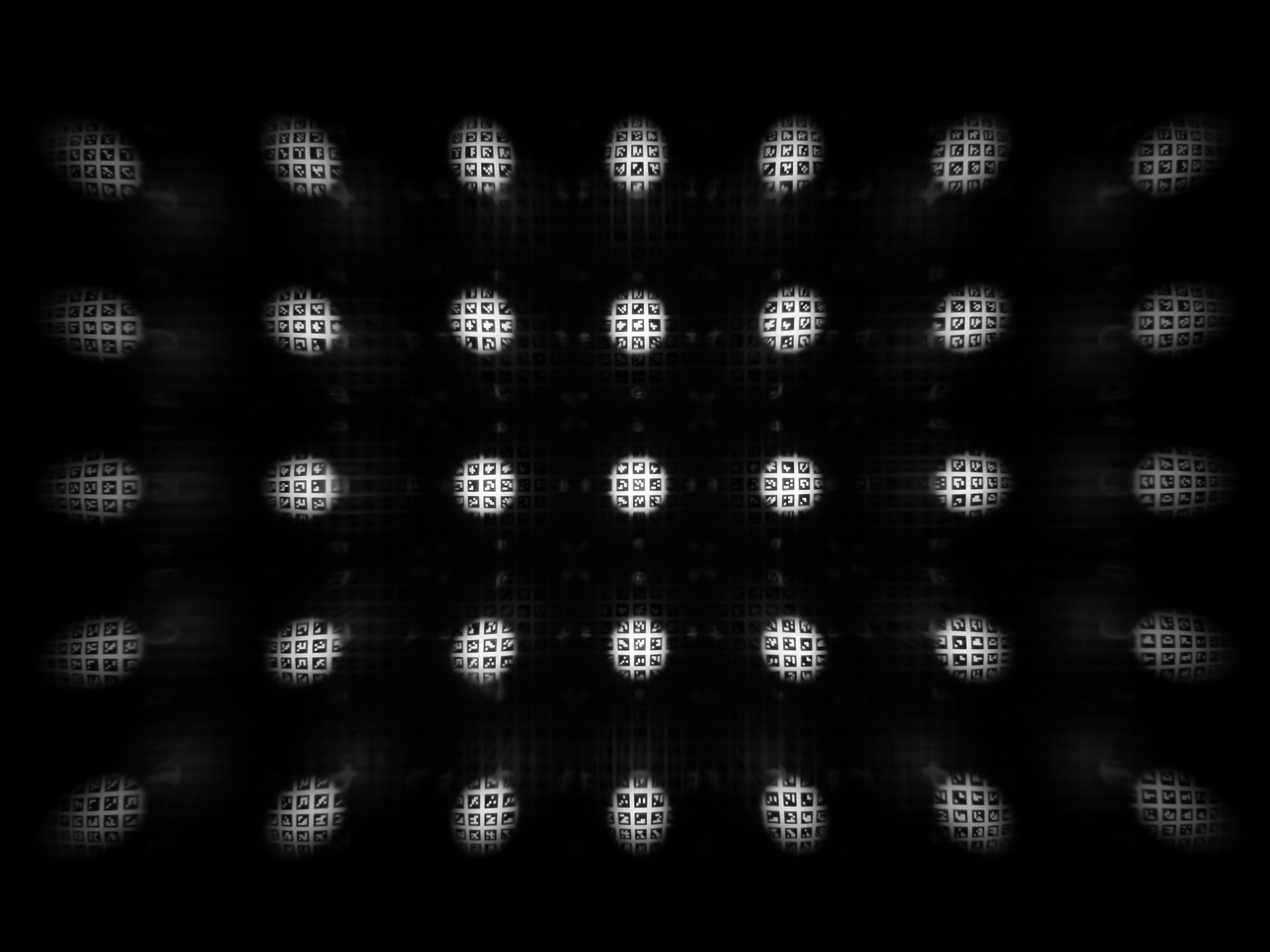

Supplement: Supplementary file 3 — Supplementary Data 1 [file 41467_2026_70967_MOESM3_ESM.zip › 3. homography estimation and registration/ArUco_01.tiff]

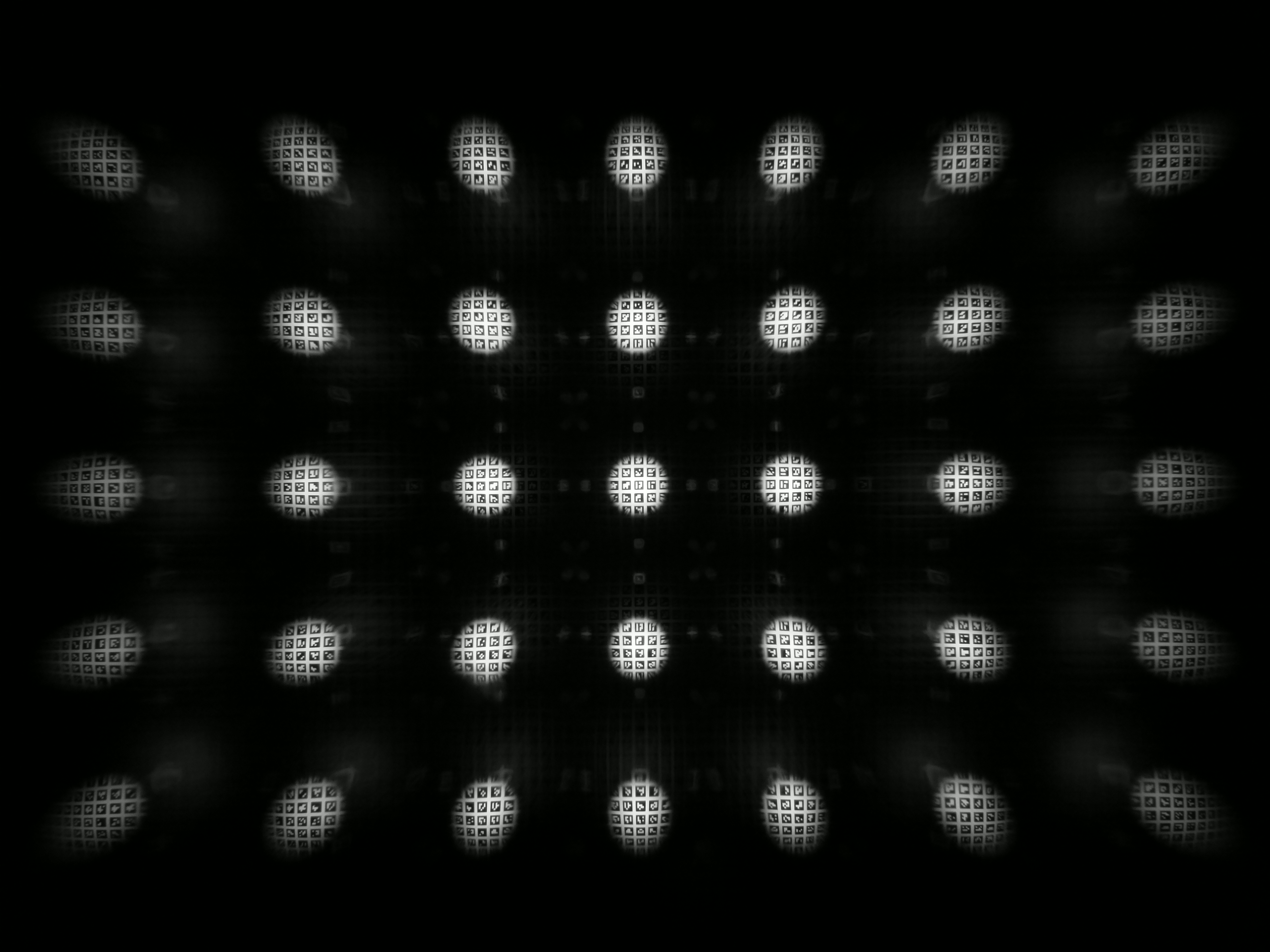

Supplement: Supplementary file 3 — Supplementary Data 1 [file 41467_2026_70967_MOESM3_ESM.zip › 3. homography estimation and registration/ArUco_02.tiff]

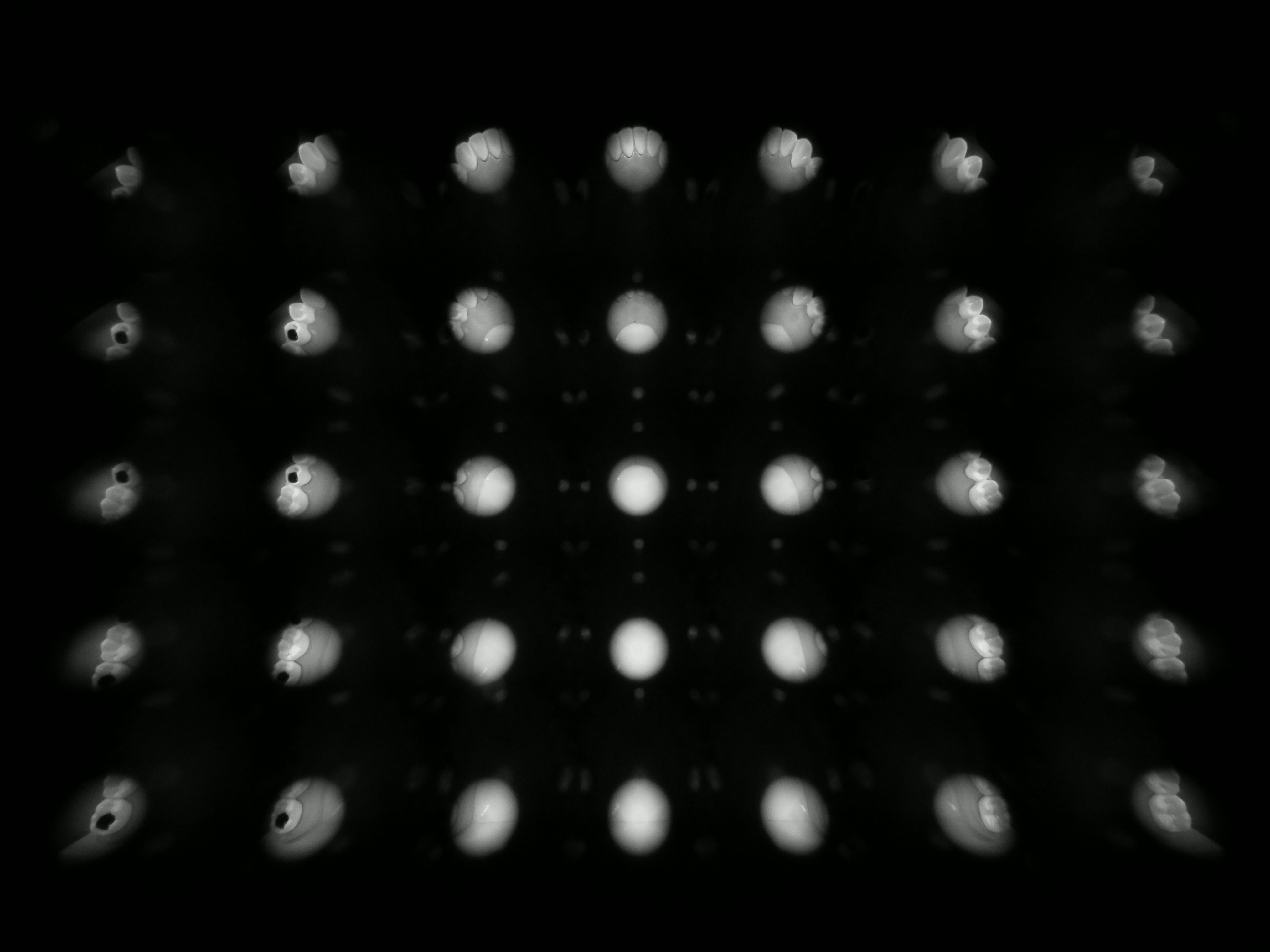

Supplement: Supplementary file 3 — Supplementary Data 1 [file 41467_2026_70967_MOESM3_ESM.zip › 4. wide fov image capture/teeth/mandibular/teeth_mandibular.tiff]

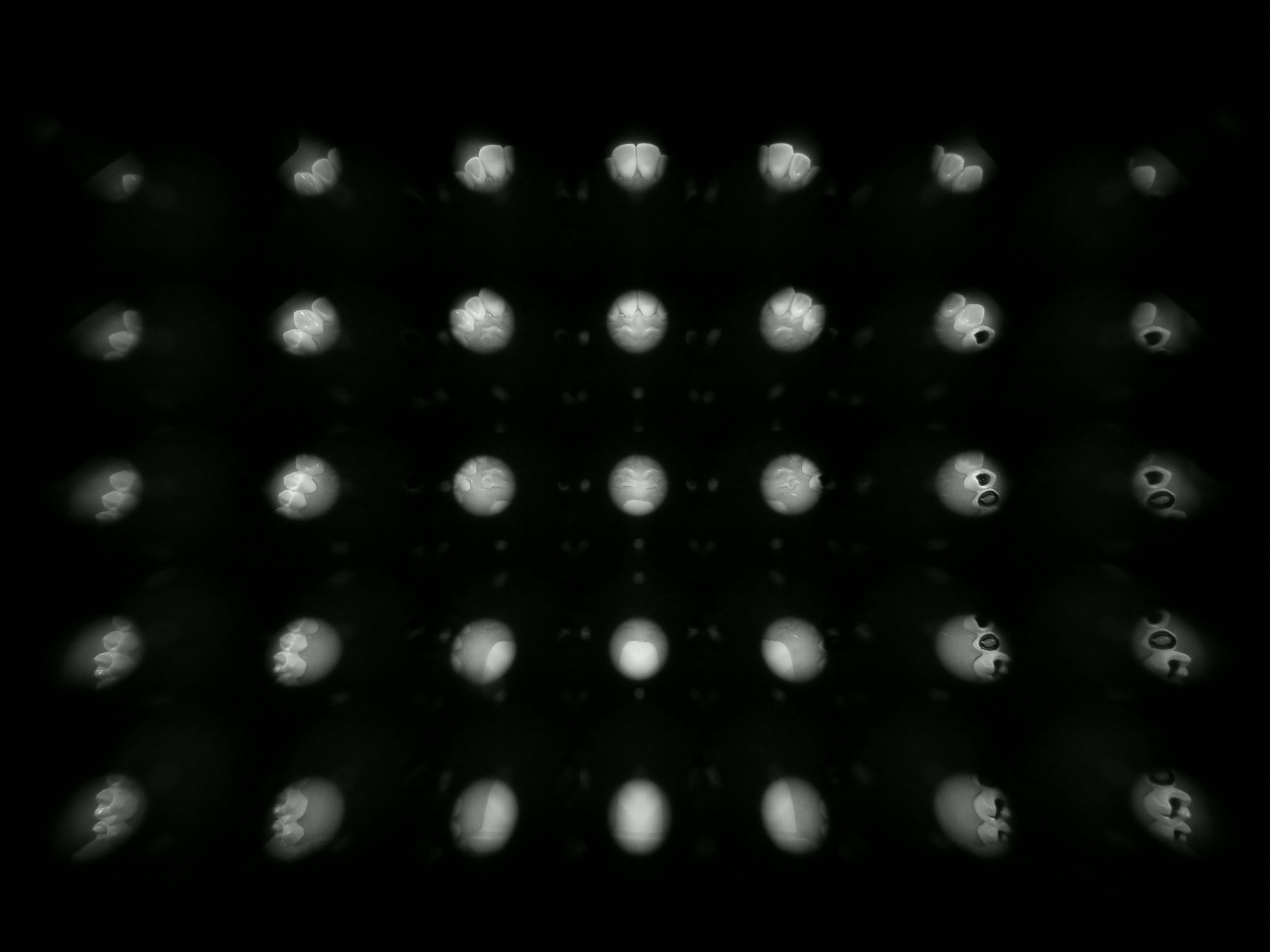

Supplement: Supplementary file 3 — Supplementary Data 1 [file 41467_2026_70967_MOESM3_ESM.zip › 4. wide fov image capture/teeth/maxillary/teeth_maxillary.tiff]
